# *Mycobacterium tuberculosis* universal stress protein Rv2623 interacts with the putative ATP binding cassette (ABC) transporter Rv1747 to regulate mycobacterial growth

**DOI:** 10.1371/journal.ppat.1006515

**Published:** 2017-07-28

**Authors:** Lisa N. Glass, Ganduri Swapna, Sivagami Sundaram Chavadi, JoAnn M. Tufariello, Kaixia Mi, Joshua E. Drumm, TuKiet T. Lam, Guofeng Zhu, Chenyang Zhan, Catherine Vilchéze, Jesus Arcos, Yong Chen, Lijun Bi, Simren Mehta, Steven A. Porcelli, Steve C. Almo, Syun-Ru Yeh, William R. Jacobs, Jordi B. Torrelles, John Chan

**Affiliations:** 1 Department of Medicine, Albert Einstein College of Medicine & Montefiore Medical Center, Bronx, New York, United States of America; 2 Department of Microbiology and Immunology, Albert Einstein College of Medicine & Montefiore Medical Center, Bronx, New York, United States of America; 3 MS & Proteomics Resource of the W.M. Keck Biotechnology Resource Laboratory, Yale University School Medicine, New Haven, Connecticut, United States of America; 4 Department of Molecular Biophysics & Biochemistry, Yale University, New Haven, Connecticut, United States of America; 5 Department of Biochemistry, Albert Einstein College of Medicine & Montefiore Medical Center, Bronx, New York, United States of America; 6 Howard Hughes Medical Institute, Albert Einstein College of Medicine & Montefiore Medical Center, Bronx, New York, United States of America; 7 Department of Microbial Infection and Immunity, College of Medicine, The Ohio State University, Columbus, Ohio, United States of America; 8 Department of Medicine, School of Stomatology and Medicine, Foshan University, Foshan, China; 9 Departments of Physiology & Biophysics, Albert Einstein College of Medicine & Montefiore Medical Center, Bronx, New York, United States of America; New Jersey Medical School, UNITED STATES

## Abstract

We have previously shown that the *Mycobacterium tuberculosis* universal stress protein Rv2623 regulates mycobacterial growth and may be required for the establishment of tuberculous persistence. Here, yeast two-hybrid and affinity chromatography experiments have demonstrated that Rv2623 interacts with one of the two forkhead-associated domains (FHA I) of Rv1747, a putative ATP-binding cassette transporter annotated to export lipooligosaccharides. FHA domains are signaling protein modules that mediate protein-protein interactions to modulate a wide variety of biological processes via binding to conserved phosphorylated threonine (pT)-containing oligopeptides of the interactors. Biochemical, immunochemical and mass spectrometric studies have shown that Rv2623 harbors pT and specifically identified threonine 237 as a phosphorylated residue. Relative to wild-type Rv2623 (Rv2623_WT_), a mutant protein in which T237 has been replaced with a non-phosphorylatable alanine (Rv2623_T237A_) exhibits decreased interaction with the Rv1747 FHA I domain and diminished growth-regulatory capacity. Interestingly, compared to WT bacilli, an *M*. *tuberculosis Rv2623* null mutant (Δ*Rv2623*) displays enhanced expression of phosphatidyl-*myo*-inositol mannosides (PIMs), while the Δ*Rv1747* mutant expresses decreased levels of PIMs. Animal studies have previously shown that Δ*Rv2623* is hypervirulent, while Δ*Rv1747* is growth-attenuated. Collectively, these data have provided evidence that Rv2623 interacts with Rv1747 to regulate mycobacterial growth; and this interaction is mediated via the recognition of the conserved Rv2623 pT237-containing FHA-binding motif by the Rv1747 FHA I domain. The divergent aberrant PIM profiles and the opposing *in vivo* growth phenotypes of Δ*Rv2623* and Δ*Rv1747*, together with the annotated lipooligosaccharide exporter function of Rv1747, suggest that Rv2623 interacts with Rv1747 to modulate mycobacterial growth by negatively regulating the activity of Rv1747; and that Rv1747 might function as a transporter of PIMs. Because these glycolipids are major mycobacterial cell envelope components that can impact on the immune response, our findings raise the possibility that Rv2623 may regulate bacterial growth, virulence, and entry into persistence, at least in part, by modulating the levels of bacillary PIM expression, perhaps through negatively regulating the Rv1747-dependent export of the immunomodulatory PIMs to alter host-pathogen interaction, thereby influencing the fate of *M*. *tuberculosis in vivo*.

## Author summary

*Mycobacterium tuberculosis* remains a significant public health burden worldwide. The tubercle bacillus can establish a clinically silent latent infection in the host, which can subsequently reactivate to cause diseases, resulting in transmission of the pathogen. We have previously shown that the *M*. *tuberculosis* universal stress protein Rv2623 has the ability to regulate mycobacterial growth and may be required for the establishment of latent infection. The present study is undertaken to better understand the mechanisms by which Rv2623 regulates *M*. *tuberculosis* growth. Our results have revealed that Rv2623 interacts with Rv1747, a putative exporter of lipooligosaccharides, to negatively regulate mycobacterial growth. We have defined the molecular elements in these two proteins that mediate their interaction. We have further shown that relative to the wild-type (WT) bacillus, an *Rv2623* null mutant (Δ*Rv2623*) exhibits a higher content of phosphatidyl-*myo*-inositol mannosides (PIMs), immunologically active molecules of the *M*. *tuberculosis* cell envelope. By contrast, Δ*Rv1747* produces less PIMs than WT. Of note, while Δ*Rv2623* is hypervirulent, Δ*Rv1747* is hypovirulent in mice. These observations link the growth-regulatory attributes of Rv2623 to the function of Rv1747, suggesting that Rv2623 may regulate *M*. *tuberculosis* growth by modulating Rv1747’s export of the immunomodulatory PIMs. Defining how Rv2623 regulates mycobacterial growth will likely provide insight into the mechanisms underlying tuberculous latency and reactivation, processes that play important roles in *M*. *tuberculosis* pathogenesis.

## Introduction

*Mycobacterium tuberculosis*, the causative agent of tuberculosis (TB), remains a global public health problem, causing, in 2015 alone, over 10.4 million new cases and 1.8 million deaths worldwide [1]. *M*. *tuberculosis* is able to establish an asymptomatic latent infection that can later reactivate to cause active diseases [2–5]. In the latently infected immunocompetent host, the lifetime risk for reactivation is 10%. In those immunocompromised, the risk for recrudescence of latent infection is 10% per year [2–5]. It has been estimated that one-third of the world’s population is infected with *M*. *tuberculosis*, and it is generally believed that the majority of these individuals harbor latent bacilli [2–5]. The latently-infected thus constitute a significant reservoir for disease reactivation and transmission. Therefore, latent TB is a major hindrance to the control and eradication of *M*. *tuberculosis*. Understanding the mechanisms that regulate tuberculous latency and reactivation may lead to the design of strategies for better TB control.

We have previously demonstrated that *M*. *tuberculosis* Rv2623, a **u**niversal **s**tress **p**rotein (USP) homolog, has the ability to regulate mycobacterial growth [6]. Results of transcriptome analysis have revealed that *Rv2623* is among the most highly induced genes when *M*. *tuberculosis* is exposed to an environment of hypoxia or nitrosative stress [7–9], conditions that tubercle bacilli are likely to encounter in an infected host [6]. *Rv2623* is also induced when *M*. *tuberculosis* is internalized by macrophages [10], in standing cultures [11], and in the lungs of infected mice during the chronic phase of tuberculous infection [7,12]. An *M*. *tuberculosis Rv2623* deletion mutant (Δ*Rv2623*) is unable to establish a chronic infection in mice and Guinea pigs, exhibiting a hypervirulence phenotype [6]. But the growth regulatory property of Rv2623 is not restricted to these *in vivo* systems. Thus, constitutive overexpression of Rv2623 in both *M*. *smegmatis* and *M*. *tuberculosis* attenuates bacillary growth *in vitro* [6]. Biochemical analysis of Rv2623 showed that it has the ability to bind ATP. Mutagenesis studies based on a 2.9 Å crystal structure yielded mutants defective in ATP binding. Analysis of these mutants revealed that the growth regulatory property of *M*. *tuberculosis* Rv2623 correlates with its ATP-binding capacity [6]. These observations prompted us to propose the possibility that *M*. *tuberculosis* Rv2623 may function as a signaling intermediate in a pathway that promotes latency [6].

Investigating the mechanisms by which this USP regulates bacillary growth, the present study provides evidence that (i) Rv2623 interacts with Rv1747, a putative ABC transporter annotated to export lipooligosaccharides [13], to negatively regulate *M*. *tuberculosis* growth; (ii) this interaction is mediated via the recognition of a conserved phosphothreonine-containing oligopeptide motif of the USP by the N-terminal FHA domain of Rv1747 (FHA I); and (iii) Δ*Rv2623*, compared to WT bacilli, exhibits a morphological phenotype that is associated with increased levels of phosphatidyl-*myo*-inositol mannosides (PIMs), immunologically active molecules that can modulate the host immune response [14,15]. Conversely, an Rv1747-deficient mutant produces lower levels of PIMs than WT *M*. *tuberculosis*. Further, while Δ*Rv2623* is hypervirulent in an infected host [6], Δ*Rv1747* is attenuated for growth *in vivo* [16,17]. Collectively, the interaction of Rv2623 with the FHA I of Rv1747 to negatively regulate *M*. *tuberculosis* growth and the divergent *in vivo* virulence and PIM phenotypes of Δ*Rv2623* and Δ*Rv1747* suggest that this USP may influence mycobacterial growth by modulating Rv1747’s functional activity, raising the possibility that Rv1747, which has been annotated as a lipooligosaccharide exporter [13], is implicated in the export of PIMs.

## Results

### Interaction of Rv2623 with the Rv1747 FHA I domain: The yeast two-hybrid study

**Fig 1:**
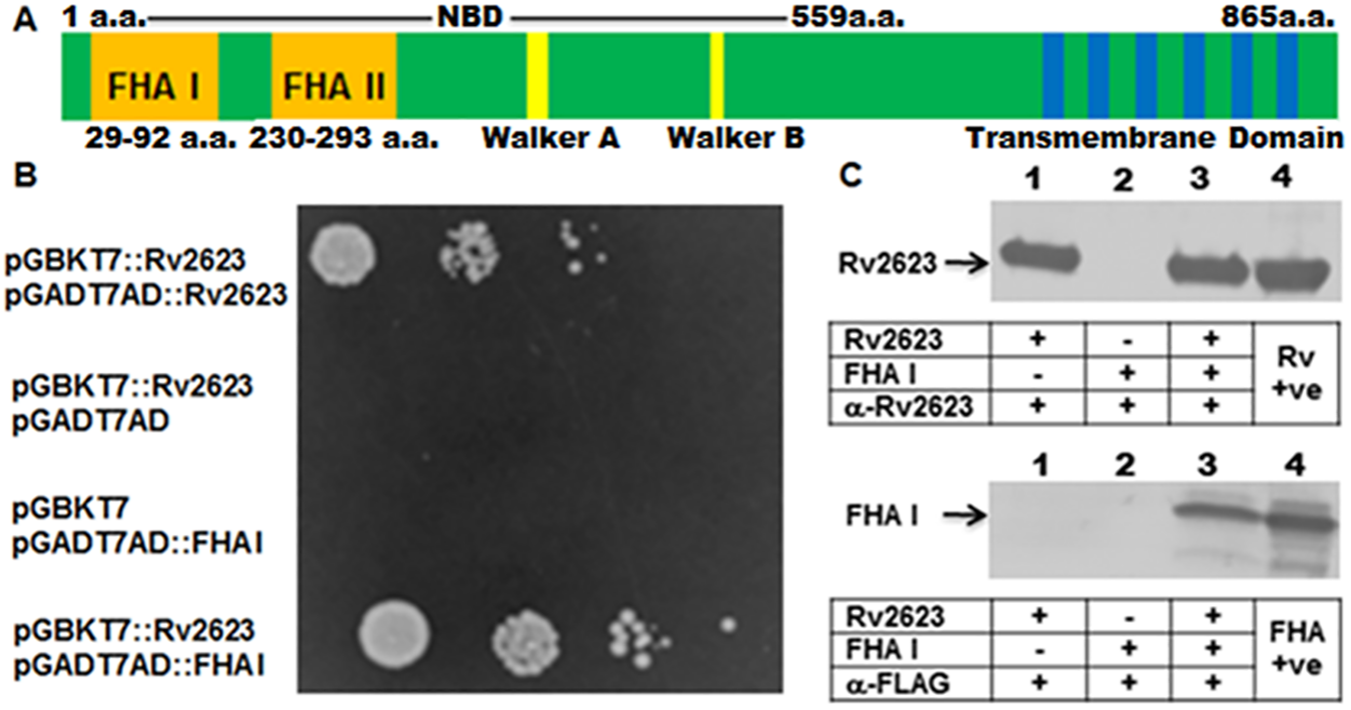
*M*. *tuberculosis* Rv2623 interacts with Rv1747. **(A)** The primary structure of Rv1747 with 2 FHA domains (Orange: FHA I & FHA II); elements typical of ABC transporters: NBD (***n***ucleoside-***b***inding ***d***omain; 1–559 amino acids; Walker A&B (Yellow; the ATP-binding domain), and transmembrane domain (Blue bars: transmembrane helices). **(B)** The GAL4-based Matchmaker Gold Yeast Two-Hybrid system was used to identify interacting partners of Rv2623, which was cloned into the pGBKT7 vector as a fusion to the GAL4 DNA-binding domain (pGBKT7::Rv2623). A DNA library of *M*. *tuberculosis* Erdman prey proteins were expressed as fusions to the Gal4 activation domain using pGADT7AD. The screen revealed that Rv2623 interacts with the N-terminal FHA I domain of Rv1747. Analysis of a re-cloned full-length Rv1747 FHA I domain validated the interaction (B, bottom panel: pGBKT7::Rv2623/pGADT7AD::FHA I). Rv2623 dimerization was exploited to serve as positive control (B, top panel; pGBKT7::Rv2623/pGADT7AD::Rv2623). pGBKT7::Rv2623/pGADT7AD and pGBKT7/pGADT7AD::FHA I represent negative controls. The interaction was further confirmed by affinity chromatography study **(C)**. Purified histidine (His_6_)-tagged Rv2623 (Rv2623) and FLAG-tagged FHA I (FHA I: first 100 amino acids of Rv1747) were expressed in *M*. *smegmatis* mc^2^155. Purified FLAG-tagged FHA I was passed over columns with or without Rv2623 immobilized onto the Nickel (Ni)-NTA resin. Western analyses of the appropriate elution fractions using anti-Rv2623 and anti-FLAG antibodies revealed that Rv2623 and Rv1747 FHA I co-eluted—upper and lower panels of lane 3 represent the results of probing eluents from column containing both (Ni)-NTA resin-immobilized (His_6_)-tagged Rv2623 and FLAG-tagged Rv1747 FHA I with anti-Rv2623 and anti-FLAG antibody, respectively—thus demonstrating interaction of these two mycobacterial components. Lane 1: upper panel and lower panel represent results of reacting eluents from column with only Rv2623 with the appropriate antibody. The upper and lower panels of Lane 2 depict reactivity of eluents from column harboring only FHA I with the appropriate antibody. Lane 4 of upper and lower panels represent recombinant Rv2623 (+ve Rv) and FLAG-FHA I (+FHA); respectively, loaded as positive controls. α-Rv2623 and α-FLAG: anti-Rv2623 and anti-FLAG antibodies; respectively. Arrows indicated the molecular weight of His-tagged Rv2623 (~32.31 kDa)) and FLAG-tagged Rv1747 FHA I (~12 kDa; expressed as the first 100 amino acids of Rv1747).

Signaling pathways often mediate their biological functions through interactions of elements involved in the cascade [18–20]. Given the possibility that Rv2623 may be an intermediate in a signaling pathway that promotes persistence [6], we sought to identify its interacting partners. Indeed, evidence exists that various bacterial USPs interacts with specific interactors to regulate physiologically relevant processes [21,22]. Using the yeast two-hybrid screen with Rv2623 as bait, we have identified a set of *M*. *tuberculosis* proteins that interact with this USP, among which is the N-terminal FHA I of Rv1747, the focus of the present study (Fig 1A and 1B). The rationale for focusing on the Rv1747 FHA I derives from the roles of forkhead-associated domains in mediating protein-protein interaction to facilitate a broad range of vital biological processes including signal transduction, transcription, cell cycle regulation, and protein transport [23–27]. Rv1747 is a putative ATP-binding cassette (ABC) transporter that harbors two FHA domains [13] (Fig 1A). The screen has identified a fragment spanning amino acids (a.a.) 46 to 142 of Rv1747, which harbors the majority of N-terminal FHA I (a.a. 29 to 92), to be an interactor of Rv2623 (Fig 1A and 1B). Yeast two-hybrid experiments using a re-cloned full-length Rv1747 FHA I domain validated its specific interaction with Rv2623 (Fig 1B).

### Interaction of Rv2623 with the Rv1747 FHA I domain: The affinity chromatography study

To further validate the interaction between Rv2623 and the Rv1747 FHA I domain, affinity chromatography experiments were conducted (Fig 1C). For these studies, purified recombinant histidine (His_6_)-tagged Rv2623 (His-Rv2623) and FLAG-tagged FHA I (FLAG-FHA I) expressed in *M*. *smegmatis* mc^2^155 [28] via a pSD series acetamide-inducible expression system were used [29,30]. FLAG-FHA I was expressed as a tagged peptide spanning the first 100 a.a. of Rv1747 (Fig 1A). Western blot analyses of the various flow-through, wash, and elution fractions collected in the affinity chromatography studies using anti-FLAG and anti-Rv2623 antibodies revealed that Rv2623 and Rv1747 FHA I co-eluted, demonstrating the interaction of these two mycobacterial proteins (Fig 1C). This interaction was similarly and consistently observed when the affinity chromatography experiments employed recombinant Rv2623 derived from the previously described *E*. *coli*-based pQE80L-*Rv2623* expression construct [6], and cMyc-FHA I harbored in the first 120 a.a. of Rv1747 and expressed in the LIC (***l***igation ***i***ndependent ***c***loning) vector pMCSG7, as well as anti-His antibodies for the detection of Rv2623 (S1 Fig). Of note, results obtained from affinity chromatography experiments showed that Rv2623 does not interact with a pSD vector-expressed recombinant C-terminal Rv1747 FHA II domain (S2 Fig). Together, these results, in conjunction with that of the yeast two-hybrid experiments, strongly suggest that Rv2623 interacts specifically with the Rv1747 FHA I in the *in vitro* systems studied.

### Bioinformatic analyses of the feasibility of *M*. *tuberculosis* Rv1747 FHA I to interact with conserved phosphothreonine-binding motifs of its interactors

**Fig 2:**
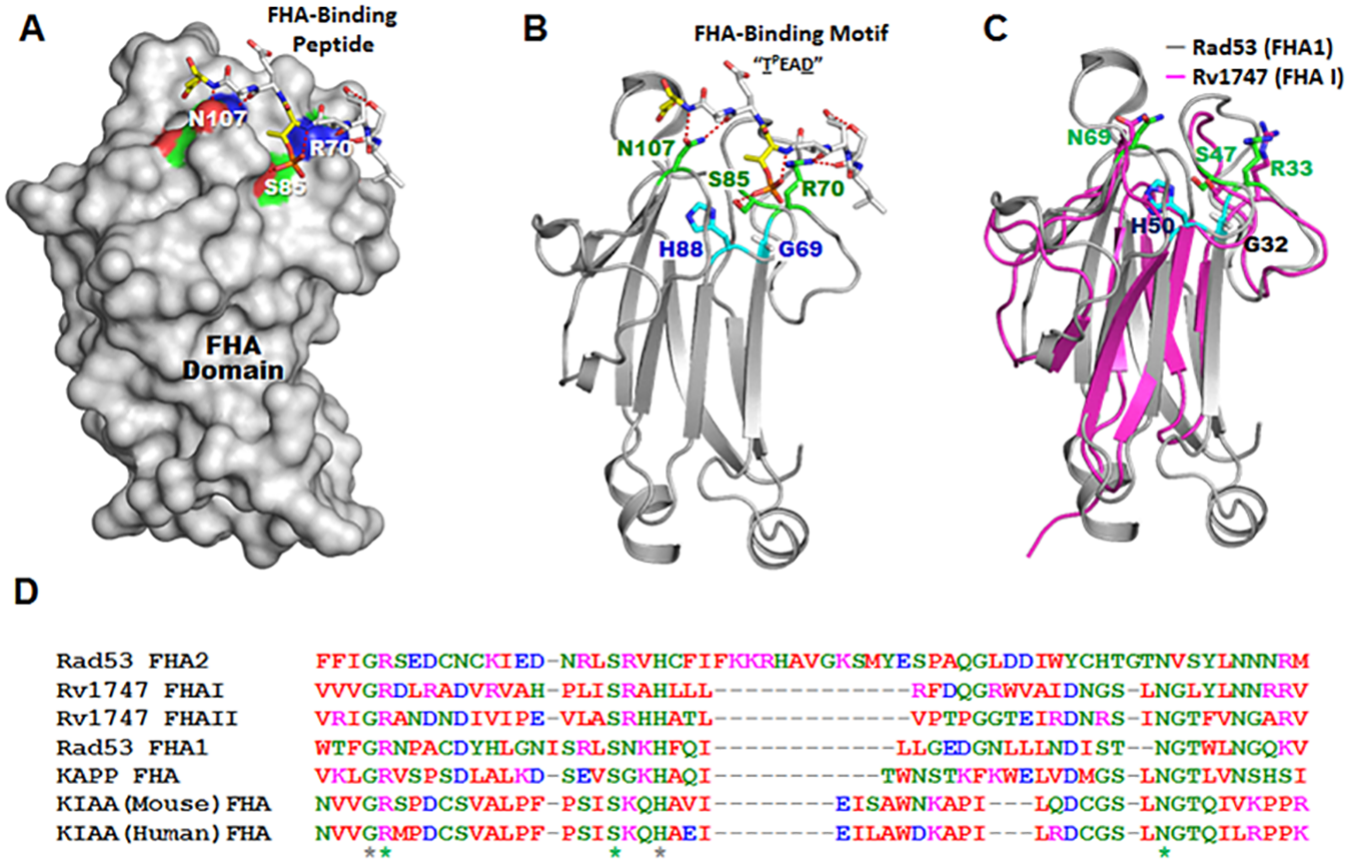
Superposition of *S*. *cerevisiae* Rad53 FHA domain with Rv1747 FHA I. **(A)** PyMol (www.pymol.org) depiction of the surface of the *S*. *cerevisiae* Rad53 FHA based on solved structures: R70, S85, and N107, the three residues in the conserved FHA domain region that have been shown to play significant roles in mediating interaction with the phosphothreonine (pT)-containing peptide motif (pTEAD) [24,32,33], are located on the surface of the yeast Rad53 FHA domain. **(B)** Ribbon diagram (www.pymol.org) of Rad53 FHA domain demonstrating interaction between R70, S85, and N107 of Rad53 with the pTEAD residues of its interacting partner’s conserved FHA-binding motif [24,32,33]. **(A&B)** H88 and G69 residues of the Rad53 FHA domain, which play a role in stabilizing the interaction between the Rad53 FHA domain and its interacting partner [24,32,33], are non-surface located. **(C)** The homology model of Rv1747 FHA I domain was generated via the M4T server ver 3.0 [34] based on comparative modeling using a combination of 2 templates (PDB codes 2LC1 and 1UHT). The homology model of the Rv1747 FHA I domain was then superimposed onto the Rad53 FHA domain [24,32,33] using Pymol (www.pymol.org): Note the superposition of conversed amino acids shown in Rad53 (N107, H88, S85, G69, R70) to play important roles in recognizing the pT-containing motif (pTEAD) [24,32,33] with the corresponding residues of Rv1747 FHA I domain (N69, H50, S47, G32, R33). **(D)** Alignment of various FHA domains with the prototypic Rad53 FHA1 and FHA2 has revealed near complete match of spacing between the conserved residues of Rad53 FHA1, known to participate in the interaction with the phosphorylated FHA domain-binding motif of its interacting partner (G69, R70, S85, H88, N107), with those of the *M*. *tuberculosis* Rv1747 FHA I domain (G32, R33, S47, H50. N69), except for one amino acid difference between Rad53 R70/S85 and the Rv1747 R33/S47 spacing. KAPP: kinase associated protein phosphatase of *Arabidopsis thaliana* [72]; KIAA: also known as KIAA0710/NFBD1 (nuclear factor BRCT domain 1) [73,74]. Note the highly conserved N, S, R (green asterisks: surface location) and H and G (Grey asterisks: non-surface location) residues.

The FHA domain, originally discovered in forkhead-type transcription factors [31], exists in a wide variety of proteins to mediate diverse functions via protein-protein interactions [23–27]. A characteristic property of FHA domains is that these interactions are based on recognition of a phosphorylated threonine (pT) residue of the binding partner [24,32,33]. One prototypic FHA domain that has been examined extensively is that of the *Saccharomyces cerevisiae* Rad53 protein kinase (Fig 2A and 2B). The Rad53 FHA1 mediates its functions in regulating cell cycle and DNA repair by binding to a pT residue that is embedded within a conserved oligopeptide motif [24,32,33]. Structural analysis of the Rad53 FHA1 has revealed that specific amino acids critical to the interaction with the pT motif are all located in the conserved region of this modular phosphopeptide recognition domain: Gly69, Arg70, Ser85, His88, and Asn107 (Fig 2A, 2B and 2D), with Asn107 and Arg70 implicated to play a role in interacting with the conversed phosphopeptide backbone. There is also evidence that Arg70, together with Ser85, play a role in hydrogen-bonding with the phosphorylated threonine of the conserved phosphothreonine-binding motif (Fig 2A and 2B) [24,32]. Parenthetically, the three amino acids critical in mediating binding with the conserved phosphopeptide motif (Asn 107, Arg70, and Ser85) are located on the surface of Rad53 FHA1 [24,32,33] (Fig 2A and 2B). The other two highly conserved non-surface FHA1 residues, Gly69 and His88, are thought to stabilize the structure of the binding site [24,32,33] (Fig 2A and 2B). The homology model of the FHA I domain of Rv1747 was generated utilizing the M4T server ver 3.0 [34] based on comparative modeling using a combination of 2 templates (PDB codes 2LC1 and 1UHT). The homology model of the Rv1747 FHA domain was then superimposed onto the Rad53 FHA domain [24,32,33] using Pymol (www.pymol.org) and displayed as shown in Fig 2C. This modeling studies of Rv1747 FHA I domain revealed a tertiary structure with 11-stranded β sandwich similar to that of the *S*. *cerevisiae* Rad53 protein kinase [24,32,33]. The conserved Gly, Arg, Ser, His, and Asn that play an important role in mediating the interaction of Rad53 with the pT motif are all present in Rv1747 FHA I (Gly32, Arg33, Ser47, His50, Asn69) with almost precisely matched spacing except for a one-amino acid difference between Rad53 FHA1 Arg70/Ser85 and Rv1747 FHA I Arg33/Ser47 (Fig 2C and 2D). In addition, tertiary structure modeling has revealed complete superposition of all the conserved a.a. between the *S*. *cerevisiae* Rad53 FHA1 and Rv1747 FHA I (Fig 2C and 2D). These results strongly support the feasibility of this *M*. *tuberculosis* forkhead-associated domain to interact with phosphorylated threonine-containing peptides. This observation prompted the initiation of study to determine whether putative phosphorylatable threonines are present in Rv2623.

### Rv2623 harbors threonine residues that can potentially mediate interaction with FHA domains

**Fig 3:**
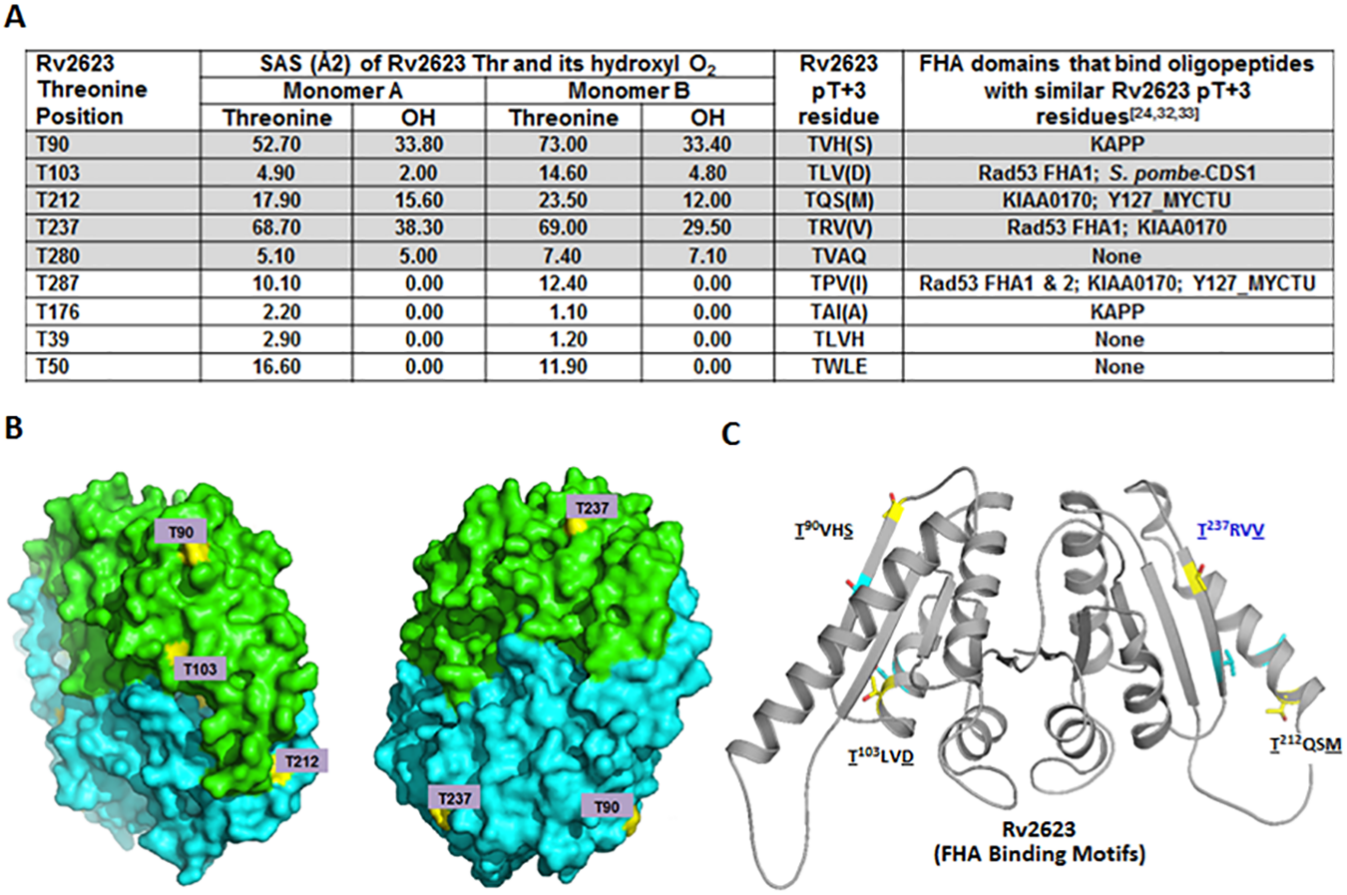
The Rv2623 threonine residues. **(A)** The solvent accessible surface (SAS) of threonine residues and the corresponding OH group in each monomer of the dimeric Rv2623 was calculated based on the crystal structure of the USP (PDB ID 3CIS) [6] using AREAIMOL program from CCP4 suite [35,36]. Among the 9 Thr residues, 5 have solvent accessible hydroxyl oxygen atoms (Shaded: T90, T103, T212, T237, T280). T90, T103, T212, and T237 also have a previously reported phosphorylation motif concerning the pT+3 residues (bracketed): pTXX(S) for T90, pTXX(D) for T103, pTXX(M) for T212, and pTXX(V) for T237 [24,32,33]. KAPP: kinase associated protein phosphatase of *Arabidopsis thaliana* [72]; KIAA: also known as KIAA0710/NFBD1 (nuclear factor BRCT domain 1) [73,74]; CDS-1: Checkpoint DNA synthesis protein kinase [75]; Y127_MYCTU: Cy1A11.16C [32], *M*. *tuberculosis* GarA [tuberculist.epfl.ch]. **(B)** The surface of the Rv2623 protein is displayed using PYMOL (www.pymol.org). The subunits A and B are colored in blue cyan and green respectively. The solvent accessible Threonine residues (T90, T103, T212, T237) are colored in yellow. **(C)** PYMOL display of a ribbon representation of an Rv2623 monomer based on previously solved structure [6] depicting the position of the four solvent accessible threonine residues with the corresponding pT+3 residues that have been shown to promote interaction with FHA domains [24,32,33]. The threonine and the pT+3 residues are labeled in yellow and cyan, respectively.

Solvent accessibility analyses based on the crystal structure of Rv2623 revealed that the hydroxyl groups of five of the nine threonine residues of the USP studied are solvent-accessible and therefore potentially phosphorylatable [6,35,36] (Fig 3A and S3 Fig). The surface locale of these five threonine residues is further confirmed by Pymol display of Rv2623 (www.pymol.org; Fig 3B). Therefore, we examined the structure for additional elements described as part of the conserved pT peptide motif signature contributing to interactions with FHA domains. We focused on the amino acid situated three residues from the corresponding phosphorylatable threonine toward the C-terminus (pT+3), because it has been observed, based on studies derived from peptide library screening experiments involving a subset of known FHA domains, that specific pT+3 residues contribute to mediating the interaction of this phosphoprotein-recognizing module with phosphorylated threonine-containing oligopeptides by modulating binding and binding selectivity [24,32,33]. Sequence analysis revealed that four of the five Rv2623 threonines whose hydroxyl group is solvent-accessible (T90, T103, T212, T237) have residues in the pT+3 position that match those found in pT-containing peptides with known FHA domain-binding specificity (Fig 3A–3C and S3 Fig) [32].

### Rv2623 is post-translationally modified and harbors phosphorylated threonines

**Fig 4:**
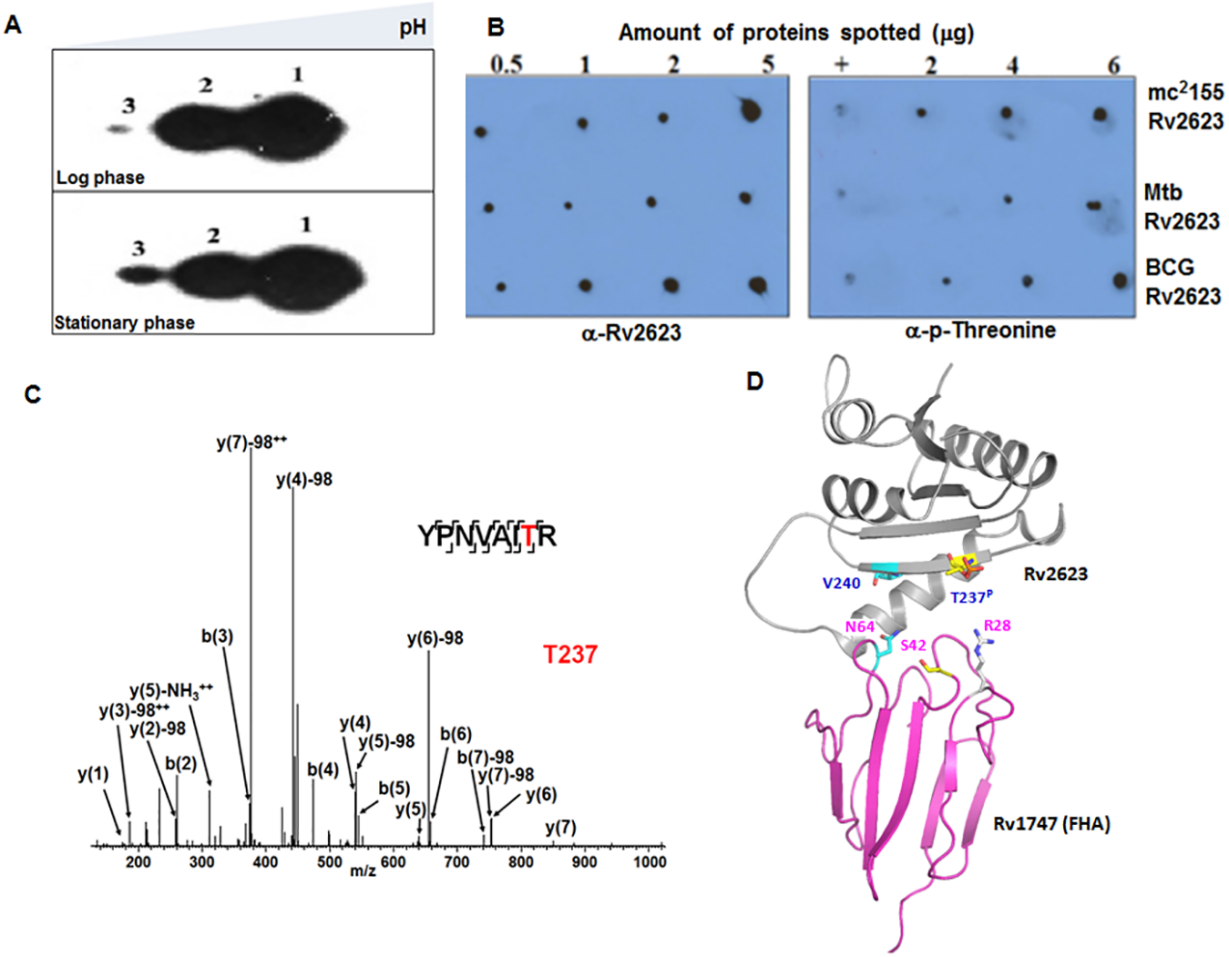
Rv2623 is post-translationally modified and phosphorylated at T237. **(A)** Immunoblot of 2D-gel electrophoretically-resolved BCG lysates with anti-Rv2623 monoclonal antibody revealed three isoforms with differing isoelectric pH values, thus providing evidence for post-translational modification of the USP; **(B)** Dot-blot analysis of affinity-purified Rv2623 from *M*. *smegmatis* mc^2^155 (Top panel); *M*. *tuberculosis* Erdman (Mid panel), and *M*. *bovis* BCG (Bottom panel) demonstrating immunoreactivity with an anti-pT antibody. “+” indicate positive pT control; soy bean trypsin inhibitor serves as negative controls (S9 Fig). **(C)** Mass spectrometry-based phosphomapping of Rv2623. The sequence coverage of the protein was ~90%. Manual examination of the appropriate MS/MS spectra was conducted to verify the phosphopeptides identified via software programs. The graph shown represents MS/MS spectra of m/z 507.24(2+) corresponding to peptide spanning amino acid residues 231–238 of Rv2623. Labelled are b- and y- fragment ions produced from Collisional Induced Dissociation (CID) in an LTQ-Orbitrap Elite LC MS/MS instrument. Phosphorylation on T237 of Rv2623 was determined based on the MS/MS fragmentation patterns; in particular are the observed loss of phosphoric acid (indicated as "y(*n*)-98Da") for fragment ions y(3), y(4), y(5), y(6), y(7), and b(7) under CID conditions [76]. The "^**++**^" sign indicates that the assigned fragment ion is doubly charged. The graph shown represents results derived from analysis of *in vitro* phosphorylated *M smegmatis-*expressed recombinant Rv2623. Analysis of vitro phosphorylated *E*. *coli-*expressed recombinant Rv2623 yielded the same results. **(D)** Molecular docking was employed to depict the interaction of the pTRVV-containing FHA domain-binding motif of Rv2623 with Rv1747 FHA I [38] (https://life.bsc.es/servlet/pydock/home/). This was performed with the [amino acids 152 to 294] domain of Rv2623 (with T237 phosphorylated) on the [amino acids 1 to 90] domain of Rv1747 FHA I, using PyDockWeb, with the d(N64-V240) constrained. The phosphate was built into T237 by the PyTMs plugin in Pymol prior to the docking. The PDB code of Rv2623 is 3cis. The structure of the FHA domain of Rv1747 is taken from Fig 2 (B and C). The Threonine residue and the pT+3 residues in the binding motifs of Rv2623 are labeled in yellow and cyan, respectively.

The above-described experiments provide strong evidence that *M*. *tuberculosis* Rv1747 FHA I can interact with Rv2623 through recognition of and binding with potentially phosphorylatable threonine residues in the context of a conserved phosphopeptide motif including the pT+3 residues (Figs 1–3). To further evaluate the interaction of these two mycobacterial components, we investigated whether Rv2623 is phosphorylated. Lysates of BCG which encodes an Rv2623 orthologue that is 100% identical to the *M*. *tuberculosis* counterpart [tuberculist.epfl.ch], were resolved by two-dimensional (2-D) gel electrophoresis and probed using a monoclonal antibody specific for Rv2623. This revealed the USP to be detected as three isoforms (Fig 4A), which have comparable approximate molecular mass, but differ based on their isoelectric points, with a shift toward the acidic direction, suggesting that Rv2623 can be post-translationally modified via phosphorylation. Further, Rv2623 affinity-purified from *M*. *tuberculosis* and BCG lysates is reactive with antibodies relatively specific for phosphorylated threonines using both a mouse monoclonal antibody and a rabbit polyclonal antibody (Fig 4B). Similarly, affinity purified *M*. *smegmatis-*expressed recombinant Rv2623 is also reactive with the anti-phosphothreonine monoclonal antibody (Fig 4B). Together, these results suggest that Rv2623 potentially can be phosphorylated at threonine residues, thus further supporting the possibility that a specific pT-containing peptide of this USP interacts with the FHA I domain of Rv1747.

### Identification of potentially phosphorylated threonine residues of Rv2623

Results of 2-D gel electrophoretic analyses of Rv2623 and its reactivity with anti-phosphothreonine antibodies (Fig 4B) led us to initiate studies to directly examine if any of the threonine residues of Rv2623 can be phosphorylated. For these studies, recombinant Rv2623 purified from *M*. *smegmatis* or *E*. *coli* that had undergone an *in vitro* phosphorylation reaction with recombinant *M*. *tuberculosis* protein kinase G (PknG) was subjected to mass spectroscopic analysis. In pilot experiments, PknG was found to be among a set of serine/threonine protein kinases (STPKs) capable of phosphorylating Rv2623 to varying degrees. This observation is in agreement with a previous report noting that *M*. *tuberculosis* STPKs are relatively promiscuous in terms of substrate use [37]. PknG was subsequently used for our studies because of its availability (gift of Dr. J. Blanchard, Albert Einstein College of Medicine). The kinased samples were resolved by sodium dodecyl sulfate-polyacrylamide (SDS-PAGE) gel electrophoresis, transferred onto nitrocellulose membrane and probed with anti-phosphothreonine and anti-Rv2623 monoclonal antibodies to evaluate for the presence of pT residues in the USP. The results revealed that Rv2623's reactivity to anti-phosphothreonine monoclonal antibody increases with time as the kinase reaction progresses (S4 Fig). For phosphomapping, the band corresponding to phosphorylated Rv2623 was excised from a Coomassie-stained gel containing electrophoretically-resolved kinasing reaction, washed, destained and then subjected to in-gel trypsin digestion. Phosphorylated species in the digest were then TiO_2_-enriched and analyzed by Liquid Chromatography-Tandem mass spectrometry (LC-MS/MS) to identify specific phosphorylated residues. This phosphomapping study identified the Rv2623 threonine at position 237, one of the solvent accessible residues that also has a conserved pT+3 amino acid known to facilitate binding of FHA domains, as a phosphorylatable residue (Fig 4C). Having demonstrated that the *M*. *tuberculosis* Rv1747 FHA I possesses structural attributes and conserved determinants that mediate interactions with interactor proteins, and that the signature forkhead-associated domain-interacting phophothreonine oligopeptide (together with the appropriate pT+3 residue) exists in Rv2623, in silico docking analysis was conducted [38]. Results of the analysis further support the interaction of these two mycobacterial components (Fig 4D).

### Analysis of the role of Rv2623 T237 in regulating mycobacterial growth

**Fig 5:**
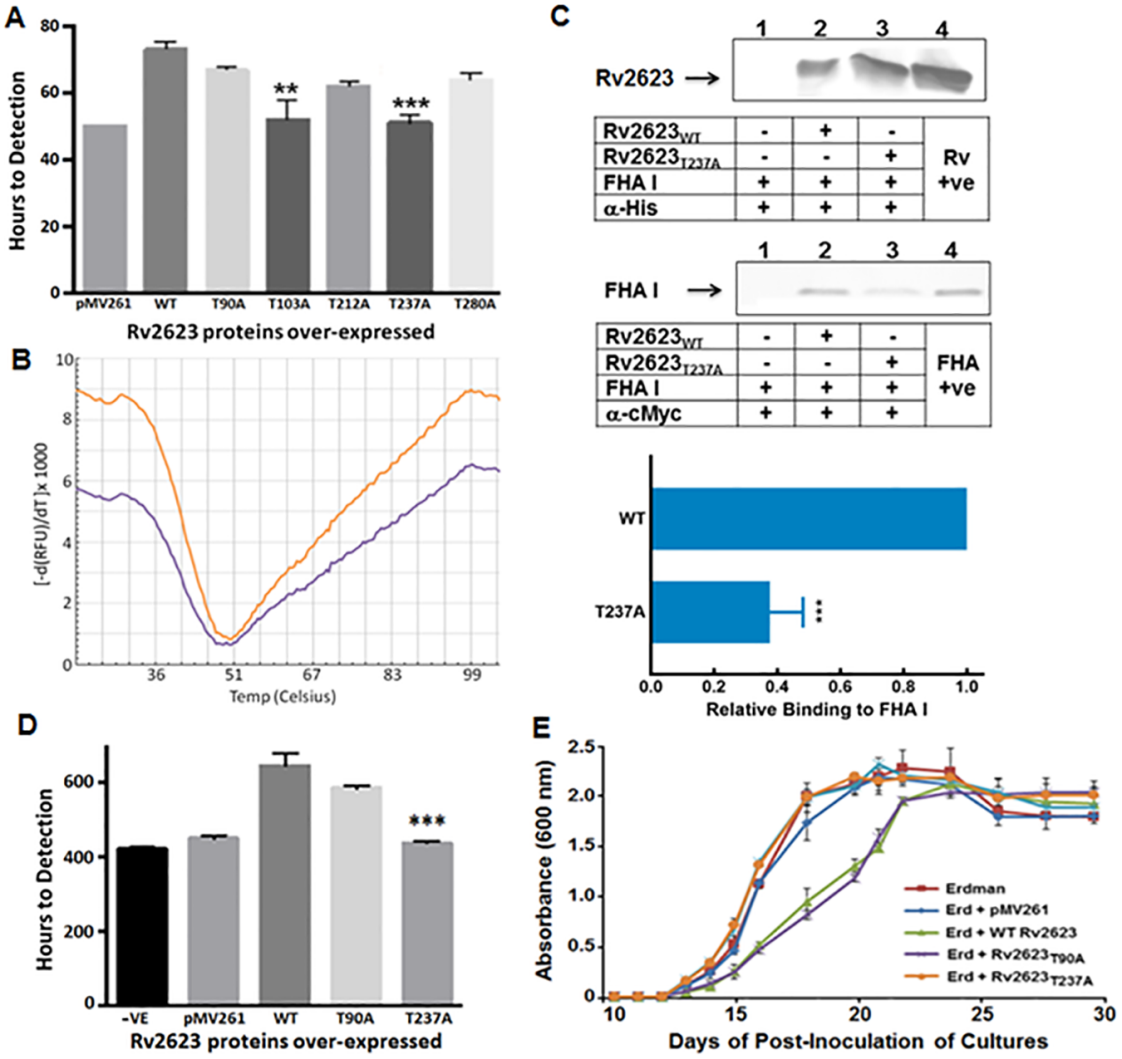
T237 of Rv2623 plays an important role in the interaction between the USP and Rv1747 FHA I to regulate mycobacterial growth. **(A)** Various T→A mutants of *M*. *tuberculosis* Rv2623 were overexpressed in *M*. *smegmatis*, and growth of the recipient cells monitored in the BACTEC 9000MB system in triplicates [6]. The time to detection reflects the rate of bacterial growth. pMV261: *M*. *smegmatis* harboring the pMV261 vector containing no Rv2623 constructs; WT: *M*. *smegmatis* overexpressing WT Rv2623 protein. The results are representative of three independent experiments. **p<0.01 (Student's *t-*test; WT vs. T103A); ***p<0.001 (Student's *t-*test; WT vs. T237A). **(B)** The melting temperature of Rv2623_WT_ (Orange) and Rv2623_T237A_ (Purple) are comparable. **(C)** These affinity chromatography studies, set up as described in Fig 2, used purified His_6_-tagged Rv2623_WT_ (Rv2623) and His_6_-tagged Rv2623_T237A_ (Rv2623_T237A_) derived from the pQE80L system; and cMyc-tagged FHA I (designated FHA I: first 120 amino acids of Rv1747) expressed via LIC vector pMCSG7. Purified FHA I was passed over columns with or without Rv2623_WT_ or Rv2623_T237A_ immobilized onto the Nickel (Ni)-NTA resin. Western blot analyses of the appropriate elution fractions were conducted using anti-His and anti-cMyc antibodies. Lane 1: negative controls–column without any Rv2623 proteins. Lane 2 and Land 3: the columns harbored Rv2623_WT_ and Rv2623_T237A_; respectively, as well as FHA I—eluents probed with the appropriate antibody revealed that relative to WT Rv2623 protein, the capacity of Rv2623_T237A_ to bind Rv1747 FHA I is diminished. Lane 4 represents positive controls for His_6_-tagged Rv2623 (Rv +ve), and cMyc-tagged FHA I (FHA +ve); respectively. The Western blot shown is representative of three independent experiments. The blue bar graph depicts densitometric analysis of the relative binding or Rv2623 proteins to Rv1747 FHA I. Error bar: standard deviation; ***p < 0.0005. Arrows indicate the molecular weight of His-tagged Rv2623_WT_ and Rv2623_T237A_ proteins (~32.31 kDa) and cMyc-tagged Rv1747 FHA I domain (~14.3 kDa; expressed as the first 120 amino acids of Rv1747). **(D)** The ability of Rv2623_WT_ to retard growth in recipient bacterial cells upon overexpression (via the multi-copy pMV261 vector) in *M*. *tuberculosis* Erdman is reversible with a T237A mutation of Rv2623, as assessed by the BACTEC 9000MB. “–ve”: untransformed *M*. *tuberculosis* Erdman; “pMV261”: Erdman transformed with pMV261 alone. ***p<0.001 (Student's *t-*test; comparing T237A with WT). **(E)** The growth-regulatory attribute of Rv2623_WT_ and the various T→A mutants in recipient cells upon overexpression in virulent *M*. *tuberculosis* Erdman was assessed by monitoring OD_600 nm_ in supplemented 7H9 Middlebrook medium. The results presented above are representative of three independent experiments. Error bars: Standard deviations.

The identification of a phosphorylatable threonine in Rv2623 with a pT+3 residue that can potentially mediate an interaction with Rv1747 FHA I (Figs 3 and 4) prompted studies designed to determine the significance of T237 in the growth-regulatory function of Rv2623. Because the phosphomapping experiment revealing that T237 is phosphorylatable used *in vitro* phosphorylated Rv2623 protein (Fig 4), we investigated, in addition to T237, the potential contributions of the other four solvent-accessible threonines of Rv2623 (T90, T103, T212, and T280 (Fig 3A)) to the growth-regulatory function of the USP. All solvent-accessible threonines of Rv2623 were individually mutagenized to alanine to yield five T→A mutants. The ability of the five mutants to attenuate growth in *M*. *smegmatis* mc^2^155 upon pMV261-based overexpression in recipient cells was evaluated using the *in vitro* BACTEC system as previously described [6]. The results of these studies revealed that, relative to the WT Rv2623, the ability of the Rv2623_T103A_ and Rv2623_T237A_ mutants to attenuate mycobacterial growth when overexpressed in *M*. *smegmatis* mc^2^155 was significantly reversed (Fig 5A). By contrast, overexpression of the Rv2623_T90A_, Rv2623_T212A_, and Rv2623_T280A_ mutants in *M*. *smegmatis* mc^2^155 displayed growth attenuating capacity comparable to that of the Rv2623_WT_. These results suggest that of the five solvent-accessible threonines, T103 and T237 are the two residues that most likely contribute significantly to the growth-regulatory property of Rv2623. Expression study, however, revealed that while the levels of overexpression of T237A mutant and WT Rv2623 proteins in *M*. *smegmatis* were comparable, Rv2623_T103A_ was poorly expressed for reasons that are currently unknown. This latter result has led us to focus solely on role of Rv2623 T237 in regulating mycobacterial growth (Fig 5A). The observation that the melting temperatures of Rv2623_WT_ (41.63°C) and Rv2623_T237A_ (42.96°C) are comparable effectively excludes the possibility that the loss of growth-regulatory ability of these threonine mutants is due to protein misfolding or instability (Fig 5B).

### The ability of Rv2623 to regulate mycobacterial growth correlates with its ability to interact with Rv1747 FHA I and requires Rv2623 T237

The fact that mutating the phosphorylatable T237 to a non-phosphorylatable alanine results in the loss of the ability of Rv2623 protein to attenuate growth upon overexpression in *M*. *smegmatis* strongly suggests that this threonine residue plays a significant role in regulating mycobacterial growth in a phosphorylation-dependent manner. This, together with the above-described evidence supporting the ability of Rv2623 to interact with the phosphopeptide-recognizing FHA I of Rv1747, has prompted us to conduct affinity chromatography experiments to examine the capacity of Rv2623_T237A_ to interact with Rv1747 FHA I. The results of these studies using purified differentially-tagged recombinant Rv2623_WT_, Rv2623_T237A_, and Rv1747 FHA I revealed that, relative to the WT Rv2623, the capacity of the T237A mutant USP to bind the *M*. *tuberculosis* Rv1747 FHA I, is significantly reduced (Fig 5C). Of note, although results derived from the affinity chromatography experiments for the study of protein-protein interaction are not quantitative, the incomplete ablation of the Rv2623-Rv1747 FHAI interaction (Fig 5C) upon T→A mutation of Rv2623 raises the possibility that additional factors may, in concert with T237 phosphorylation, regulate the interaction between these two mycobacterial proteins. Collectively, these results strongly suggest that mycobacterial growth can be regulated through the interaction of the phosphorylated T237 residue of Rv2623 and the FHA I of Rv1747.

To examine whether the Rv2623-Rv1747 interaction plays a role in regulating growth in virulent *M*. *tuberculosis* as demonstrated in *M*. *smegmatis*, the ability of Rv2623_T237A_ mutant protein to attenuate growth in the Erdman strain upon overexpression was examined using the *in vitro* BACTEC system [6]. As in the *M*. *smegmatis* study, the T237A but not T90A mutation of Rv2623 significantly reversed the ability of WT USP to regulate *M*. *tuberculosis* growth (Fig 5D). *M*. *tuberculosis* overexpressing the Rv2623_T237A_ mutant protein exhibited growth kinetics comparable to that of the WT Erdman strains lacking pMV261 or harboring the vector control (pMV261 with no *Rv2623* sequence). Rv2623_WT_, Rv2623_T90A_, and Rv2623_T237A_ proteins were overexpressed to comparable levels in *M*. *tuberculosis* Erdman via pMV261 (S5 Fig), thus excluding the possibility that the observed inability of the T237A mutant protein to retard growth of the tubercle bacillus is due to inadequate expression. The growth regulatory property of Rv2623_WT_, Rv2623_T90A_, and Rv2623_T237A_ was further examined in *M*. *tuberculosis* by growth curve analysis monitoring OD_600nm_ (Fig 5E). In agreement with the BACTEC study, growth curve analysis also indicated attenuated growth in strains expressing Rv2623_WT_ and Rv2623_T90A_, while that expressing Rv2623_T237A_ mutant grew at a rate similar to Erdman and the Erdman+pMV261 control (Fig 5E). Taken together, these results strongly suggest that T237 of Rv2623 is critical for the growth-regulatory function of this USP through mediating, in its phosphorylated form, the interaction with Rv1747: Binding of pT237-containing Rv2623 to the FHA I domain of Rv1747 negatively regulates *M*. *tuberculosis* growth.

### *M*. *tuberculosis* Rv2623- and Rv1747-deficient mutant exhibit opposing and aberrant cell wall expression of phosphatidyl-myo-inositol mannosides (PIMs)

**Fig 6:**
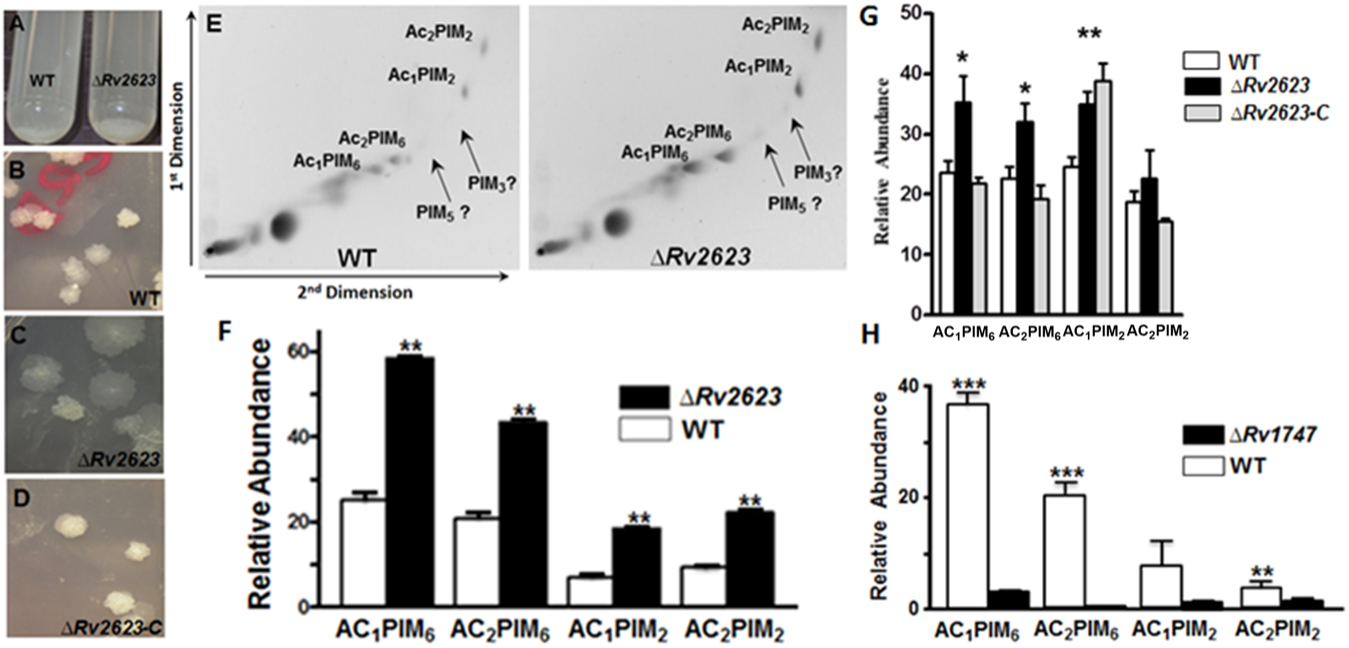
PIM expression by *M*. *tuberculosis* Δ*Rv2623 and* Δ*Rv1747*. **(A)** *M*. *tuberculosis* WT and Δ*Rv2623* in stationary phase cultures in supplemented Middlebrook 7H9 medium were let to stand for 10 min (Left tube: *M*. *tuberculosis;* Right tube Δ*Rv2623*). The deletion mutant displays a sedimentation phenotype distinct than that of WT. **(B,C)** *M*. *tuberculosis* Δ*Rv2623*, plated on 7H10 agar medium supplemented with OADC, displays a colony morphology different than that of WT. **(E)** Analysis of *M*. *tuberculosis* lipid extracts by 2-dimensional thin layer chromatography (TLC) revealed that compared to WT, Δ*Rv2623* expresses higher level of PIMs; results of densitometric quantification is depicted in **(F).** In a separate set of experiments designed to evaluate whether the PIM phenotype of Δ*Rv2623* is Rv2623-specific, bacteria were grown in Middlebrook 7H11 + OADC-supplemented agar at 37°C, 5% CO_2_ and harvested 12 days post-plating, and lipid extraction and 2D-TLC were carried out as previously published [77]. Complementation of **(B-D)** the colony morphology phenotype and **(G)** PIM phenotype of Δ*Rv2623* via the integrating vector pMV306. The PIM results are representative of 2–3 biological samples, each analyzed in three independent experiments. **(H)** Δ*Rv1747* expresses PIMs at a level lower than that of WT. Error bars: Standard deviation. *p<0.05; **p<0.01; ***p<0.001.

During the course of manipulation of the *M*. *tuberculosis* Rv2623-deficient mutant, it was observed that this strain displays a sedimentation phenotype when grown in Middlebrook 7H9 (M7H9) medium with Tween 80 (Fig 6A). Under this culture condition, WT Erdman and Δ*Rv2623* grow with similar kinetics monitored over a 2 week-period [6]. Upon standing of suspension of individual 10-day-old bacterial cultures, the Δ*Rv2623* strain formed a loosely-packed, fluffy sediment compared to WT (Fig 6A). In addition, when plated onto M7H10 agar, these two strains display distinct colony morphotypes, with the Rv2623-deficient strain forming colonies with an apparently smoother, less ruffled appearance (Fig 6B and 6C), which is restored to that of WT with complementation, thus demonstrating Rv2623-specificity of this phenotype (Fig 6B–6D). This observation, together with the functional assignment of Rv1747 as a putative exporter of lipooligosaccharides, prompted us to begin characterizing cell envelope components of *M*. *tuberculosis* Δ*Rv2623*. We initiated studies to evaluate the expression of PIMs, one of the most abundant and bioactive glycolipid families in the *M*. *tuberculosis* cell wall [14,15]. Two-dimensional thin-layer chromatography (TLC) analyses of total polar lipid extracts (normalized by total protein content) revealed that the Δ*Rv2623* mutant displays a PIM profile distinct from that observed in WT Erdman (Fig 6E and 6F). Specifically, Δ*Rv2623* expresses higher amounts of PIMs, particularly tri- and tetra-acylated PIM_2_ and PIM_6_, compared to WT bacilli (Fig 6E & 6F). In a separate and independent series of experiments, Rv2623-specificity for the hyper-producing phenotype for all the PIM types examined was demonstrated by complementation—using the integrating pMV306 construct that expresses Rv2623 under the control of its native promoter [6]—except for AC_1_PIM_2_ (Fig 6G). The apparent unique attribute of this latter PIM species is unclear. Given the observation that Rv2623 interacts with Rv1747 to negatively regulate *M*. *tuberculosis* growth, the enhanced PIM levels of Δ*Rv2623* suggest that Rv2623 may affect *in vivo M*. *tuberculosis* growth by modulating the production of PIMs, raising the possibility that Rv1747 (which has been annotated to be an exporter of lipooligosaccharides [13]), could be involved in PIM transport. Consequently, we evaluated the amount of PIMs in an Rv1747-deficient mutant. The results have revealed that *M*. *tuberculosis* Δ*Rv1747* is a hypo-producer of PIMs relative to WT Erdman (Fig 6H and S6 Fig). This latter observation lends support to the notion that Rv1747 can indeed be involved in the transport of PIMs, thereby influencing their levels in the *M*. *tuberculosis* cell wall.

## Discussion

Given the remarkable number of people world-wide estimated to harbor dormant tubercle bacilli [2–5], which physiological state renders treatment challenging [39], tuberculous latency and reactivation poses a formidable problem for the effective control of *M*. *tuberculosis*. The mechanisms by which tuberculosis latency and reactivation are regulated remain incompletely defined [2–5]. We have previously reported that *Rv2623*, which encodes a USP and is highly induced by hypoxia and nitrosative stress, can regulate bacillary growth *in vitro* and *in vivo* [6]. Importantly, virulent *M*. *tuberculosis* deficient in Rv2623 fails to establish a chronic persistent infection and is hypervirulent in an infected host [6]. This latter observation suggests that Rv2623 plays a role in regulating *in vivo M*. *tuberculosis* growth, particularly in the context of tuberculous latency [6].

The present study seeks to understand the mechanisms by which Rv2623 regulates *M*. *tuberculosis* growth. The results, derived from yeast two-hybrid screen, affinity chromatography studies, crystallographic, as well as bioinformatics and modeling analyses [6,24,32,33], have provided evidence that this USP interacts with Rv1747, a putative ABC lipooligosaccharide exporter [13], to negatively regulate *M*. *tuberculosis* growth in concert. This interaction is mediated via the recognition by the Rv1747 FHA I domain, an Rv2623 conserved oligopeptide motif harboring a phosphorylated threonine at position 237, as assessed by mass spectrometry-based phospho-mapping study. Compared to the WT USP, the interaction of Rv1747 FHA I domain with a mutant Rv2623 protein, which T237 has been mutated to a non-phosphorylatable alanine (Rv2623_T237A_), is significantly compromised. Significantly, *in vivo* analyses of specific Rv2623 mutants have shown that the interaction of the USP with Rv1747 has functional consequences: relative to Rv2623_WT_, the growth regulatory capacity of the mutant Rv2623_T237A_ is markedly attenuated. Together, these results have provided strong evidence that specific elements of Rv2623 and the FHA I domain of Rv1747 mediate the interaction between these two mycobacterial components to negatively regulate *M*. *tuberculosis* growth in a phosphorylation-dependent manner.

In support of the observation on Rv2623-Rv1747 interaction, evidence exist that bacterial USPs mediate biological functions in concert with interacting molecules. For example, *Escherichia coli* UspC functions as a scaffolding protein of the KdpD/KdpE two-component salt sensing signaling pathway, and via phosphorylation-dependent mechanisms, regulates the expression of the high-affinity K^+^ transporter KdpFABC [21]. There is evidence that the *Halomonas elongate* USP TeaD may regulate the activity of the ectoine transporter TeaABC to maintain osmotic equilibrium [22]. The KdpD/KdpE and TeaD studies thus provide evidence for the interaction between bacterial USP and transporters, supporting our observation that the *M*. *tuberculosis* USP Rv2623 interacts with the putative ABC transporter Rv1747.

*M*. *tuberculosis* Rv1747, a 92-kDa integral membrane protein, has been annotated as an ABC transporter [13]. The Rv1747’s nucleotide-binding domain (NBD) and its membrane spanning domain (MSD), two major components of a typical ABC transporter, are fused [13,40]. This structural organization suggests that the transporter operates as a homodimer [13]. Although its function remains to be formally characterized, Rv1747 harbors elements strongly suggesting that it is an ABC transporter [40]. In addition to the NBD and MSD domains, Rv1747 carries the Walker A and B motifs, which make up the ATP-binding pocket of ABC transporters [40]. Rv1747 also harbors the ABC transporter family signature sequence, a characteristic 12-residue segment located between the two Walker motifs, and the 6-amino acid Linton and Higgins motif downstream of Walker B [40]. *In vitro* studies have revealed that the Rv1747 NBD displays ATPase activity [41]. A unique feature of Rv1747 is the presence in the NBD of two FHA domains, phosphopeptide-recognizing signaling modules that mediate diverse biologically important processes via protein-protein interactions [23–27]. Of the various *M*. *tuberculosis* ABC transporters, Rv1747 is the sole FHA domain-containing member [13]. And Rv1747, one of the 6 FHA domain-containing *M*. *tuberculosis* proteins (S7 Fig), is the only one in this group to harbor two such domains [23,26].

The FHA domain, together with the bacterial counterparts of eukaryotic STPKs, is an important component of reversible phosphoregulation pathways that mediates a wide range of biological processes [23–27]. Characterization of FHA-containing *M*. *tuberculosis* proteins including EmbR (Rv1267c) [42–46], FhaB (Rv0019c) [47], and GarA (Rv1827, glycogen-accumulation regulator A) [43,48–50] has revealed that these molecules, together with other mycobacterial components and STPKs, play important roles in regulating biologically highly significant processes, including synthesis of cell wall components, cell division, drug resistance, virulence levels, and metabolism. The observation that Rv2623 interacts with Rv1747 to regulate bacterial growth adds to the gravity of *M*. *tuberculosis* FHA domain-containing components. Rv1747 is required for optimal survival of *M*. *tuberculosis* in macrophages, dendritic cells, and in mice [16,17], and this virulence attribute is dependent on its phosphoregulation by PknF [16,17]. Thus, a Δ*Rv1747 M*. *tuberculosis* mutant, while exhibiting no *in vitro* growth phenotype, is hypovirulent in mice [16,17]. This latter hypovirulence phenotype is in stark contrast to the *in vivo* hypervirulence of Δ*Rv2623* [6]. Given the observation that Rv2623 and Rv1747 interact to negatively regulate *M*. *tuberculosis* growth, the opposing *in vivo* virulence phenotype of Δ*Rv2623* and Δ*Rv1747* suggests that the USP may modulate the growth of the tubercle bacillus *in vivo* by attenuating the function of the putative transporter. This scenario, together with the divergent PIM phenotype of the Rv2623- and Rv1747-deficient mutants (with Δ*Rv2623* and Δ*Rv1747* being a hyper- and hypo-producer of PIMs; respectively), raises the possibility that the putative ABC transporter Rv1747 serves to transport PIM(s), and that these glycolipids may, at least in part, impact mycobacterial growth *in vivo* through their immune-regulatory attributes [14,15].

Lending credence to the above notions, there exist precedents that ABC transporters function as exporters of phospho- and glycolipids [51–53] and that protein phosphorylation serves as a regulatory mechanism of this transport system [52,54–56]. As well, it has been reported recently that among the most differentially up-regulated genes in an *M*. *tuberculosis* Δ*Rv1747* strain, relative to WT bacilli, are *iniA* and *iniB* [57], members of the *iniBAC* operon which are induced by a variety of agents that inhibit mycobacterial cell wall synthesis [58,59]. Thus, the differentially enhanced expression of genes of this operon in Δ*Rv1747* could reflect the bacterial reaction to certain dysregulated cell wall biosynthesis pathways. This latter idea is consistent with the diminished level of expression in Δ*Rv1747* of PIMs, molecules that are important components of the *M*. *tuberculosis* cell envelope, which also serve as precursors for the synthesis of lipomannan (LM) and mannose-capped lipoarabinomannan (LAM), the latter a major mycobacterial surface lipoglycan that is essential in the tubercle bacillus [14,15,60]. Collectively, the *ini* operon observations, together with annotation of Rv1747 as an ABC transporter that exports lipooligosaccharides, and the diametrically opposed growth and PIM phenotypes of Δ*Rv2623* and Δ*Rv1747*, have provided evidence suggesting that Rv1747 may function as a PIM exporter, perhaps translocating certain member(s) of this family of glycolipids from the cytoplasmic side to the periplasmic side of the plasma membrane [14,60].

The enzymes for PIM biosynthesis and the subcellular locales in which PIMs are produced are incompletely defined [14,60]. Evidence exists, however, that synthesis of PIM is compartmentalized, with the lower (PIM_1/2_) and higher (PIM_5/6_) order PIMs produced in the cytosolic and periplasmic side of the inner membrane, respectively [14,60]. This paradigm suggests that translocation of PIMs (PIM_2_, PIM_3_, and/or PIM_4_) across the inner membrane must occur [14]. Nonetheless, bona fide transporter(s) of PIMs, their substrate specificity, and how many there are, are unknown [14]. Results of the present study suggest that Rv1747 may function as a PIM transporter. Due to the essentiality of PIM_1_, PIM_2_, and PIM-derived LM/ManLAM for *M*. *tuberculosis* survival, the biosynthesis of PIMs (including their translocation across the inner membrane) and related lipoglycans is likely complex and may involve compensatory and/or redundant mechanisms [14]. Indeed, the results of a study on PIM_4_ channeling protein LpqW suggest that compensatory mechanisms are in place to ensure the maintenance of adequate levels of the essential LAM at the expense of higher order PIMs [61,62]. It is possible that this apparent LAM-preserving attribute of *M*. *tuberculosis* could be operative in the PIM-hypoproducing Δ*Rv1747*. This would explain the results of a report noting that a Δ*Rv1747* strain, despite being a hypo-producer of PIMs (the precursors for LAM biosynthesis), the mutant displays WT level of LAM/ManLAM, notwithstanding the use of a non-quantitative immuodetection approach and the lack of evaluation of PIM expression by the deletion mutant [57].

**Fig 7:**
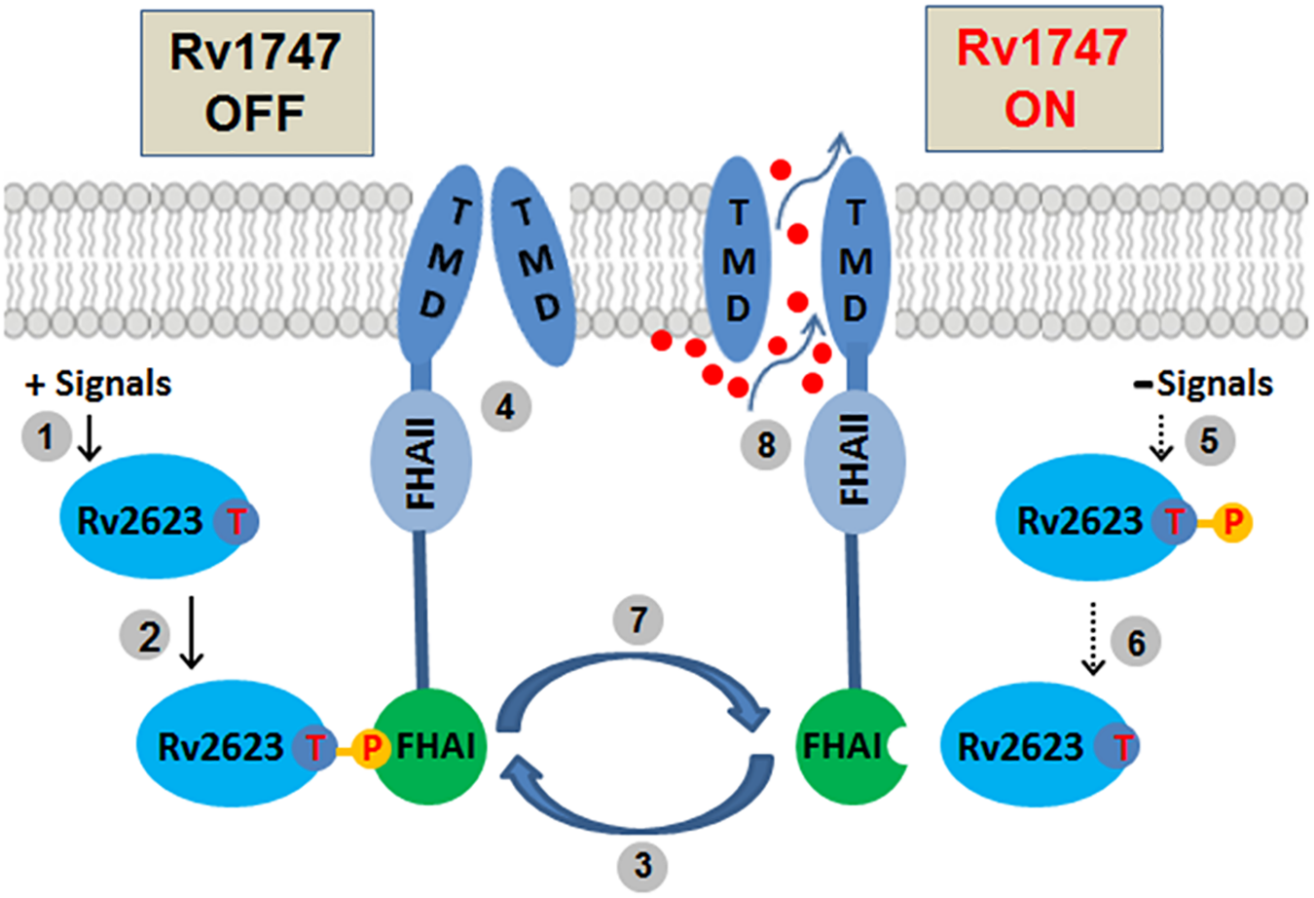
Schematic of the regulation of Rv1747 putative PIM transport by Rv2623. In response to certain signals encountered in the host **(1)**, the threonine residue of Rv2623 of *M*. *tuberculosis* (a universal stress protein) at position 237 (red “T” in purple sphere) is phosphorylated **(2).** This results in the formation of a conserved phosphothreonine (pT237)-containing motif that enables the engagement of Rv2623 with the FHA I domain of Rv1747 **(3).** This interaction negatively modulates the function of the putative transporter Rv1747, turning it off **(4)**. In the absence of the signals operative in step **(1)**, or in the presence of additional signals **(5)**, dephosphorylation of the phosphorylated Rv2623 occurs **(6)**, leading to disengagement of Rv2623 from Rv1747 FHA I **(7)**. This disengagement releases the inhibitory effect of the phosphorylated Rv2623, allowing Rv1747 to transport the putative substrates PIMs **(8)**. Whether PIMs are the substrates for Rv1747 remains to be proven. The signals that induce the phosphorylation of Rv2623 are presently unclear–potential candidates include hypoxia and nitrosative stress, as well as nutritional restriction. The nature of the kinase that phosphorylates Rv2623 *in vivo* is also unknown. The Rv1747 is depicted in its monomeric form except for its transmembrane domain (TMD) for clarity. Rv2623 tethered to an orange circle with a red P represents the phosphorylated form. Red “T” in purple sphere: T237. Small red spheres represent the substrates transported by the Rv1747 transporter.

The present study has provided evidence that *M*. *tuberculosis* Rv2623 negatively modulates the transport function of Rv1747 to regulate mycobacterial growth through phosphorylation-dependent mechanisms (Fig 7). Further, the opposing PIM expression phenotype of the Δ*Rv2623* and Δ*Rv1747* mutants suggests that Rv1747 may be a transporter for PIMs, immunologically active molecules that have been shown *in vitro* to impact *M*. *tuberculosis-*host interaction to influence the immune response [14,15]. The immune-regulatory properties thus could contribute, at least in part, to the ability of the Rv2623-Rv1747 interaction to regulate *M*. *tuberculosis* growth in the host by influencing the anti-tuberculous response [14,15]. It is also plausible that the altered levels of expression of these important cell enveloped glycolipids can have an intrinsic effect on mycobacterial growth. These two possibilities are not mutually exclusive. PIMs are downregulated in stationary phase [14], and it has recently been observed that the production of PIMs by *M*. *tuberculosis* is enhanced during infection in primary human macrophages (S8 Fig). This latter result reinforces the concept that the tubercle bacillus can adapt to environmental signals during infection by modulating the cell envelope, including lipid components [63–67]. Whether the level of PIMs in the *M*. *tuberculosis* cell envelope can influence *in vivo* mycobacterial growth and virulence is currently unclear; that it can is supported by our findings that the PIM-hyperproducing Δ*Rv2623* is hypervirulent *in vivo* and is unable to establish a chronic infection, while Δ*Rv1747*, a hypoproducer of PIMs, is attenuated for growth in mice [16,17].

Much remains to be learned regarding the precise mechanisms by which Rv2623 modulates mycobacterial growth and by which this USP is regulated. The presence of three apparent Rv2623 isoforms suggests that the regulation of this protein is complex, and that phosphorylation of T237 might not be the only mode of post-translational modification involved. The phosphorylated Rv2623 that was subjected to mass spectrometric analysis was generated via an in vitro phosphorylation reaction mediated by recombinant PknG. Hence it is possible that in vivo, other threonines, in addition to T237, could serve as additional targets of phosphorylation, thus accounting for the three Rv2623 isoforms observed. It is also possible that other mode of post-translational modification is involved. Equally unclear is the mechanism underlying the previously described dependency of the *M*. *tuberculosis* growth-regulating attribute of Rv2623 on its ATP-binding capacity [6]. Is this dependency related to the Rv2623-Rv1747 interaction? Finally, the apparent function of Rv1747 as a PIM transporter and the mechanisms underlying its regulation remain to be characterized. This will likely not be a straightforward endeavor given the transmembrane nature of Rv1747. Nevertheless, the collective results of the present study have provided a framework for understanding the mechanisms by which Rv2623 interacts with the putative PIM transporter Rv1747 (Fig 7) to regulate *M*. *tuberculosis* growth, particularly in the context of tuberculous persistence, and for potentially advancing knowledge of the biosynthetic pathways of mycobacterial glycolipids and lipoglycans.

## Materials and methods

### Bacterial strains, culture conditions, and molecular cloning

Primers, oligonucleotides (IDT DNA technologies) and vectors used for generating various constructs are listed in S1 Table. *M*. *tuberculosis* strain Erdman and H37Rv and *M*. *smegmatis* mc^2^155 were cultured in supplemented Middlebrook 7H9 (M7H9) medium (Becton Dickinson, Sparks, MD) as previously described [6]. Mycobacterial strains overexpressing WT Rv2623 and various mutants of the USP were maintained in supplemented M7H9 broth containing 40 μg/ml of kanamycin. Mycobacterial growth was monitored by measuring absorbance at 600 nm or by the *in vitro* BACTEC 9000 system as previously described (Becton Dickinson, Sparks, MD) [6]. The latter approach involved inoculating stationary phase *M*. *tuberculosis* or *M*. *smegmatis* in triplicates into BD Myco/F lytic vials (final bacterial suspension of 10^4^ colony forming units (CFU) per ml) (BD Bosciences, Sparks, MD), whose liquid medium is supplemented with a compound that fluoresces as a result of oxygen depletion due to bacterial growth. The time to detection of fluorescence signals thus reflects the rate of bacterial growth.

The pSD series vectors, which direct expression of proteins of interests in *M*. *smegmatis* under the control of the acetamide-inducible promoter, were used to generate N-terminal His_6_-tagged and N-terminal FLAG-tagged recombinant Rv2623 (His-Rv2623 and FLAG-Rv2623) in mc^2^155 [29,30]. Fragments of Rv1747 representing the first 120 amino acids and harboring the FHA I domain (Fig 1A) were PCR-amplified to contain the cMyc or FLAG tag and cloned into the pSD vectors for the expression of an N-terminal cMyc-tagged FHA I (Myc-FHA I) and N-terminal FLAG-tagged FHA I (FLAG-FHA I) [29,30]. An N-terminal FLAG-FHA II (spanning amino acids 201–310) fusion (FLAG-FHA II) was similarly expressed in the pSD system [29,30]. The pQE80L (Qiagen, Inc.)-based plasmid pQE-*Rv2623* containing an N-terminal His_6_-tag-*Rv2623* fusion construct was also used for the expression of His-Rv2623 [6]. Various *M*. *tuberculosis* Rv2623 threonine residue mutants were derived from pQE-*Rv2623* via site-directed mutagenesis and similarly expressed (see below). As well, Myc-FHA I and FLAG-FHA I were also expressed in *E*. *coli* via the LIC (Ligation Independent Cloning) vector pMCSG7 [68] (pMCSG7-*His-TEV-cMyc-FHA I*; pMCSG7-*His-TEV-FLAG-FHA I*) (TEV: Tobacco Etch Virus cleavage site).

### Expression and purification of recombinant *M*. *tuberculosis* proteins

The plasmid pSD31-*Rv2623* was electroporated into *M*. *smegmatis* mc^2^155 for the expression of His-Rv2623 in this acetamide-inducible system [29,30]. Log-phase cultures were induced with acetamide (final concentration: 0.2%) overnight at 37°C with shaking. Expression of *M*. *tuberculosis* Rv2623 based on the pQE80L system was carried out as previously described, following isopropyl beta-D-thiogalactoside (IPTG; final concentration, 0.3mM) induction in transformed BL21 *E*. *coli E*. *coli* [6]. The threonine residue mutants of Rv2623 were similarly expressed. Protein purification was carried out as described with modification [6]. Auto-induction medium was used for the expression of recombinant proteins via the LIC vector pMCSG7 [68]. Cells were disrupted using hydraulic press or the Matrix B and Fast Prep apparatus (MP Biomedicals, CA) for Mycobacterium and sonication for *E*. *coli*. Clarified bacterial cell lysates were filter-sterilized and expressed proteins were purified by affinity chromatography using appropriate antibodies against specific tags or Rv2623 followed by gel-filtration chromatography as described previously [6] (S1 Text).

### Yeast two-hybrid identification of Rv2623 interacting proteins

*M*. *tuberculosis* Erdman strain chromosomal DNA and pGADT7 AD (Clontech, Mountain View, CA) cloning vector DNA were used to generate the yeast library for the yeast two-hybrid screen. Construction of the library was carried out by DNA Technologies Inc. (Gaithersburg, MD). Two *E*. *coli* libraries (> 2 x 10^6^ clones per library) were generated by cloning partially digested, size-fractionated chromosomal DNA consisting of fragments spanning 0.5 to 2.5 kb as well as those ranging from 2.5 to 5.0 kb into the BamHI site of the pGADT7AD vector. These two DNA libraries, which were used to screen for Rv2623-interacting prey proteins were cloned in pGADT7AD harboring the GAL4 activation domain. pGADT7AD has the Leu2 nutritional marker for selection in yeast. The gene for the bait protein Rv2623 was cloned into the pGBKT7 vector to generate an Rv2623-GAL4 DNA-binding domain translational fusion. pGBKT7 has the TRP1 nutritional marker for selection in yeast. The screens were conducted using Matchmaker Gold Yeast Two-Hybrid System, according to manufacturer’s instructions. To confirm the interaction between the prey hits with Rv2623, the putative interactor sequence harbored in pGADT7AD was rescued. pGADT7AD harboring DNA fragments expressing the putative interactor were then individually co-tranformed with pGBKT7 containing the full-length Rv2623 DNA into an auxotrophic reporter strain Y2H Gold (*his*^*-*^, *ade*^-^) for validation of the interaction. The positive control was Rv2623 interacting with itself, as it forms a dimer in solution [6].

### Affinity chromatography and immunoblotting experiments

*E*. *coli*- and/or *M*. *smegmatis-*expressed His-tagged full-length WT Rv2623 (Rv2623_WT_), the Rv2623_T237A_ mutant, and cMyc-tagged FHA I, and FLAG-FHA II were purified by affinity chromatography using the appropriate antibody followed by gel filtration as described for use in the affinity chromatographic study [6]. Spin columns were packed with 50 μl of Ni-NTA agarose bead slurry (~25 μl resin; Qiagen) and equilibrated with Buffer A (S1 Text). Resins in the equilibrated columns were either left untreated or allowed to react with 150 μg each of either His-tagged Rv2623_WT_ or Rv2623_T237A_, for 2 hours at 4°C. The columns were then washed to rid of unbound proteins using Buffer B (S1 Text). Appropriate columns were allowed to react with 400 μg each of recombinant cMyc or FLAG-tagged FHA I for 2 hours at 4°C and then thoroughly washed to remove unbound proteins using Buffer A and Buffer B sequentially. The columns were then eluted for bound proteins with 100 ml of Buffer C (S1 Text). The eluates were electrophoretically resolved and subjected to Western blot analysis using antibodies specific for the specific epitope tag or to Rv2623, followed by an appropriate Horseradish peroxidase-conjugated secondary antibody, and then subjected to detection by chemiluminescence (Amersham). Proteins were densitometrically quantified (S1 Text).

### Two-dimensional gel electrophoretic and Western blot analysis of *M*. *tuberculosis* Rv2623

The BioRad system was used for the 2-D gel electrophoresis analysis. To prepare samples for isoelectric focusing, 150 μg total protein (1 μg for purified protein) was processed using a ReadyPrep 2-D Cleanup Kit (Bio-Rad, Hercules, CA) according to the manufacturer’s instructions. The prepared protein (final volume: 125 μl) was allowed to react with a ReadyStrip IPG, pH range 4–7 (Bio-Rad) in a rehydration tray for ~16 hours at 20°C. The IPG strip thus prepared was subjected to isoelectric focusing for three hours (8kV for 10kvh (50μA/strip)). The focused IPG strip was equilibrated and washed according to the manufacturer’s instructions, and loaded onto a 1.5 mm-thick 14% SDS polyacrylamide gel with a 4% stacking gel, along with a Precision Plus Protein Dual Color Standard (Bio-Rad, Hercules, CA). The gel was run at 155 V until the dye front migrated to the bottom of the gel (~60 min). In some cases, the gel was then stained with Sypro Ruby (Bio-Rad, Hercules, CA) according to the manufacturer’s instructions. In other cases, the SDS gel was transferred to a PVDF membrane and analyzed for protein separation and detection of Rv2623 by standard Western blotting techniques. The PVDF membrane was reacted with a primary monoclonal antibody against *M*. *tuberculosis* Rv2623 (Advanced Immunochemicals, Inc., Long Beach, CA; 5-Rv2623-A10) followed by a secondary horseradish peroxidase-conjugated anti-mouse IgG antibody. Protein was visualized by chemiluminescence using a Super Signal West Pico Chemiluminescent Substrate (ThermoFisher Scientific, Waltham, MA) according to the manufacturer’s instructions and exposed to Kodak scientific imaging film (BioMax XAR, Eastman Kodak Company, Rochester, NY). Relative abundance of protein was quantified using ImageQuant densitometry software (Molecular Dynamics, Inc; Sunnyvale, CA). In some experiments, the PVDF membrane was also stained with Coomassie Brilliant Blue G-250 (Bio-Rad, Hercules, CA) according to the manufacturer’s instruction.

### Dot-blot analysis for Rv2623 phosphorylation

Recombinant Rv2623 derived from *M*. *smegmatis*, as well as the native USP immunoaffinity-purified from *M*. *tuberculosis* and BCG cell lysates were analyzed for phosphorylation using a dot blot assay. Briefly, increasing concentrations of Rv2623 protein derived from the various sources were spotted onto nitrocellulose membrane. Phosphothreonine (Sigma-Aldrich; St. Louis, MO) served as positive control. The membrane was allowed to dry, followed by blocking with 5% BSA in Tris-HCl (20 mM Tris-HCl; pH 7.5) containing 150 mM NaCl, 0.05% Tween 20) (TBST). The membrane was probed with anti-phosphothreonine antibodies: clone #42H4 mouse monoclonal or rabbit polyclonal antibodies (Cell Signaling Technology; Danvers, MA), washed thrice with TBST and then reacted with appropriate horseradish peroxidase (HRP)-conjugated secondary antibody (Sigma-Aldrich; St. Louis, MO). The blot was developed using ECL reagent (Amersham; Piscataway, NJ).

### Mass spectrometry analysis of Rv2623 phosphorylation

Purified recombinant Rv2623 (5 μg) [6] was subjected to *in vitro* phosphorylation by *M*. *tuberculosis* PknG as described in S1 Text. The reaction product, electrophoretically resolved in a 10% SDS PAGE, was subjected to Western blot analysis using anti-phosphothreonine antibody (Cell Signaling; Danvers, MA). In parallel, a separate gel of electrophoretically resolved reaction mixture was stained with Coomassie Blue to identify the Rv2623 band based on molecular mass. The Coomassie Blue-stained gel band harboring the phosphorylated Rv2623 protein was excised, and the sample sent in chloroform-treated microcentrifuge tubes (Eppendorf; Westbury, NY) to the Mass Spectrometry & Proteomics Resource of the W.M. Keck Foundation Biotechnology Resource Laboratory (Yale School of Medicine, New Haven, CT) for further processing and mass spectrometric analysis for phosphorylated threonine based on published protocol [69]. Briefly, the gel band was SpeedVac-dried, then solubilized in appropriate buffer. The protein sample was then subjected to dithiothreitol (DTT) reduction, IAN-mediated alkylation, and the urea content brought down to 2 M with water prior to trypsin digestion. The digested sample was acidified with 0.5% trifluoroacetic acid (TFA), 50% acetonitrile and then subjected to titanium dioxide enrichment using the Top Tips system (Glygen Corp; Columbia, MD). The resulting phosphopeptide-enriched sample, dissolved in 70% formic acid and diluted with 0.1% TFA, was then subjected to LC-MS/MS analysis using the LTQ Orbitrap Elite that is equipped with a Waters nanoACQUITY UPLC system, and which uses a Waters Symmetry C18 180 μm x 20 mm trap column and a 1.7 μm, 75 μm x 250 mm nanoACQUITY UPLC column for peptide separation. The acquired data was peak-picked and searched using the Mascot Distiller and the Mascot search algorithm, respectively; and quantitatively processed with Progenesis LCMS (Nonlinear Dynamics, LLC) as previously described [69] (S1 Text). Protein identification was achieved using Mascot search algorithm (Matrix Science; Boston, MA) as described [69] (S1 Text). Manual examination of the MS/MS spectra was conducted to verify the phosphopeptides identified via software programs.

### Generation of strains of *M*. *smegmatis* mc^2^155 and *M*. *tuberculosis* Erdman overexpressing WT Rv2623 and various Rv2623 threonine mutants

Wild-type *Rv2623*, with and without a His_6_ tag, was cloned into the pMV261 vector under the control of the hsp60 promoter using 5’-EcoRI and the 3’-HindIII sites [30]. This construct served as template for the generation of specific Rv2623 single amino acid substitution threonine mutants by mismatched PCR priming using primers designed to direct the incorporation of specific mutations into the *Rv2623* coding region following Stratagene Quikchange (La Jolla, CA) protocol as described [6]. All constructs were confirmed by sequence analysis. Appropriate constructs were transformed into *M*. *smegmatis* mc^2^155 and *M*. *tuberculosis Erdman* to generate mycobacterial strains over-expressing WT Rv2623 and the desired threonine mutants. Transformants were screened by PCR targeting the kanamycin resistance marker (for *M*. *smegmatis and M*. *tuberculosis*) and/or *Rv2623* (for *M*. *smegmatis*). PCR product from *M*. *smegmatis* was gel purified (Qiagen gel extraction kit; Valencia, CA) and the sequence of the WT and mutant *Rv2623* verified. Following sequence verification, expression of Rv2623 and its mutants in *M*. *smegmatis* was probed by Western blot, using a mouse anti-Rv2623 monoclonal antibody (Advanced Immunochemical; Long Beach, CA). To distinguish between the native and pMV261-based expression of the USP and its mutants in *M*. *tuberculosis*, a mouse anti-His tag monoclonal antibody (Sigma-Aldrich; St. Louis, MO) was used. Finally, thermal denaturation curves were determined for purified WT and mutant Rv2623 using an IQ5 Real Time PCR Detection System (Bio-Rad; Hercules, CA) following incubation with SYPRO Orange protein gel stain (ThermoFisher Scientific, Waltham, MA) as previously described [6] (S1 Text).

### Lipid analysis of *M*. *tuberculosis* strains

For analysis of PIMs located in the cell wall of *M*. *tuberculosis* strains, bacteria were harvested 12 days after inoculation on M7H11 agar plates and lysed [70]. Protein quantification was performed by the BCA method following the manufacturer’s instructions (Bio-Rad, Hercules, CA). Lysates totaling 10 mg of protein from each strain were delipidated at 37°C for 12 h using CHCl_3_:CH_3_OH (2:1, v/v) followed by CHCl_3_:CH_3_OH:H_2_O (10:10:3, v/v/v) for an additional 12 h. For 2D-TLC analyses, total crude lipids from each strain were loaded (100 μg) onto the origin of a 10 cm × 10 cm TLC silica gel 60 F_254_ aluminum plate (EMD Millipore, Temecula, CA) based upon equal amounts of protein content from the bacterial lysates and run in the first dimension using CHCl_3_:CH_3_OH:H_2_O (60:30:6, v/v/v) as a solvent system. The TLC plate was then rotated 90° to the left and run in the second dimension using CHCl_3_:CH_3_COOH:CH_3_OH: H_2_O (40:25: 3:6, v/v/v/v) as a solvent system. Plates were dried and sprayed with 10% concentrated sulfuric acid in absolute ethanol and heated at 110°C until lipid bands appeared [71]. The NIH ImageJ program (http://rsb.info.nih.gov/ij/) was used to perform PIM densitometry analysis by calculating mean spot intensities from three independent experiments.

## Supporting information

S1 Text Supplementary materials and methods (DOCX)

S1 Fig (DOCX)

S2 Fig (DOCX)

S3 Fig (DOCX)

S4 Fig (DOCX)

S5 Fig (DOCX)

S6 Fig (DOCX)

S7 Fig (DOCX)

S8 Fig (DOCX)

S9 Fig (DOCX)

S1 Table (XLSX)
