# Supplementary material for: Mycobacterium tuberculosis universal stress protein Rv2623 interacts with the putative ATP binding cassette (ABC) transporter Rv1747 to regulate mycobacterial growth
Source: PLoS Pathog. 2017 Jul 28;13(7):e1006515. doi: 10.1371/journal.ppat.1006515 (PMC5549992; doi:10.1371/journal.ppat.1006515)
Supplement: S1 Text — (DOCX) [file ppat.1006515.s001.docx]

**Supporting Information**

**S1 Text: Supplementary Materials and Methods**

**Purification of recombinant proteins.**

Bacterial cells harboring the desired expressed recombinant protein were collected by centrifugation (2,500 *g*). For the His-tagged recombinant proteins expressed in the pQE80L or pSD systems, the pellet re-suspended in lysis buffer (Buffer A (50 mM HEPES, 300 mM NaCl, 15 mM imidazole, 10% glycerol, 2 mM MgCl_2_, pH 8.0) containing lysozyme (1 mg/ml, Sigma-Aldrich; St. Louis, MO), phosphatase and protease inhibitors (Roche Diagnostics; Indianapolis, IN). Cells were disrupted using hydraulic press or the Matrix B and Fast Prep apparatus (MP Biomedicals; Santa Ana, CA) for Mycobacterium and sonication for *E. coli*. The lysates were clarified by centrifugation (10,000 *g*), filtered with 0.22-μm filter before transfer out of the BSL-3 facility to be incubated with HisPur Ni-NTA (nitrilotriacetic acid) resins (Thermoscientific; Waltham, MA) for 1 hour at 4^o^C as previously described [1]. The resins were washed with 10 column volumes of Buffer B (50 mM HEPES, 300 mM NaCl, 50 mM imidazole, 10% glycerol, 2 mM MgCl_2_, pH 8.0). His-tagged protiens were eluted with Buffer C (50 mM HEPES, 300 mM NaCl, 250-500 mM imidazole, 10% glycerol, 2 mM MgCl_2_, pH 8.0). Fractions containing the desired His-tagged protein, as determined by monitoring absorption at 280 nm, were pooled and concentrated for second step purification by gel filtration using a Superdex 200 10/300 GL column (GE Healthcare Life Sciences; Pittsburgh, PA). The Superdex column was equilibrated with 50 mM HEPES containing 50 mM NaCl, 2 mM MgCl_2_, 10% glycerol and 13 mM β-mercaptoethanol (pH 8.0) and calibrated using the following molecular mass standards: aldolase (158 kDa), bovine serum albumin (67 kDa), ovalbumin (43 kDa), chymotrypsinogen A (25 kDa) as described in the Amersham Pharmacia technical notes (GE Healthcare Life Sciences; Pittsburgh, PA). The flow rate was set to 0.15 ml/min and elution of the protein was monitored at 280 nm absorption. Protein was stored in aliquots at -80^๐^C until further analysis. Similar approach was used for the purification of cMyc- and FLAG-tagged recombinant proteins expressed via the pSD [2,3] and pQE80L (Qiagen, Inc.; Valencia, CA) systems.

For protein expression using the LIC system, the plasmid vector containing the cMyc- or FLAG-tagged Rv1747 FHA I domain (1-120 amino acids; pMCSG7-*His-TEV-cMyc-FHA I* and pMCSG7-*His-TEV-FLAG-FHA I*; respectively) was transformed into BL21 and grown in auto-induction media [4]. The protein expressed consists of an N-terminal cMyc- or FLAG-tagged-FHA I with an upstream His tag separated from cMyc or FLAG by a TEV protease cleavage site. FLAG-FHA II fusion protein was similarly produced. Bacterial lysis and protein purification using conventional Ni^2+^-NTA-based method was conducted as described above. Elution fractions were analyzed on SDS-PAGE, peak fractions were pooled and subjected to incubation with TEV protease overnight at 4°C to remove the N-terminal His tag. This fraction was further passed over Ni^2+^-NTA resins to allow binding of the His-tagged TEV protease, the TEV-cleaved-free His tags, and any uncleaved His-TEV-cMyc/FLAG-FHA I to the Ni^2+^ resin, thus enabling collection of the pure cMyc-FHA I or FLAG-FHA I that is free of His tag. The protein collected in the FT fraction was analyzed on SDS-PAGE, transferred onto nitrocellulose membrane, probed with anti-His antibody to ensure successful elimination of the His tag. Purification of the desired recombinant protein was assessed by Western blot analysis using tag-specific antibodies ( S1 Fig - FHA I). cMyc-FHA I or FLAG-FHA I protein was further purified to homogeneity by gel filtration chromatography on Superdex 200 10/300 GL column as described above.

**Affinity chromatographic study**

Ni-NTA resin (Qiagen)-packed columns were equilibrated with 10 column volumes of Buffer A. Excess buffer was removed by centrifuging at 650 *g* for 5 minutes (all subsequent centrifugation used these same parameters). Control columns harbor purified Rv2623_WT_, the Rv2623_T237A_ mutant, or FHA I alone. Experimental columns contain either a mixture of Rv2623_WT_ with FHA I or Rv2623_T237A_ with FHA I. Ni-NTA resin-packed columns were allowed to react at 4^o^C for 2 hours with 150 μg of the appropriate Rv2623 proteins (Rv2623_WT_ or Rv2623_T237A_) in Buffer A. Unbound proteins were collected as flow-through (FT1) upon centrifugation and stored at 4^○^C. The columns were loaded with 10 volumes of Buffer B. The columns were agitated for 15 minutes at 4^○^C before centrifugation to collect the wash buffer, which was stored at 4^○^C until use. Following washing, appropriate columns were allowed to react with 400 μg of c-Myc-FHA I or FLAG-FHA I at 4^○^C for 2 hours, at which time, unbound proteins were collected as flow-through (FT2) and stored at 4^○^C until use. The columns were then washed three times: first with Buffer A (W) and then twice with Buffer B. The bound proteins were eluted with 100 μl of Buffer C (E).  Samples FT2, W, and E were subjected to Western blot analysis. Protein samples were combined with Laemmli’s SDS-PAGE buffer and boiled at 100°C for 10 min. Proteins were resolved by electrophoresis using 12% Tris-tricine polyacrylamide gels (Biorad; Hercules, CA). Resolved proteins were transferred to a polyvinylidine fluoride (PVDF) membrane (Biorad; Hercules, CA) and probed with appropriate anti-His (Sigma-Aldrich; St. Louis, MO), anti-Rv2623 (Advanced Immunochemicals, Inc., 5-RV2623-A10), anti-FLAG (Sigma-Aldrich; St. Louis, MO) and anti-cMyc (Thermofisher; Waltham, MA) antibodies to detect Rv2623_WT_, Rv2623_T237A_, and FHA I. Proteins were detected with the appropriate HRP-conjugated secondary antibodies, using the Amersham ECL (**e**nhanced **c**hemi**l**uminescence) Plus Chemiluminescence kit (Amersham; Piscataway, NJ) and quantified via densitometry. Co-IP studies designed to examine the interaction between Rv2623 and Rv1747 FHA II domain were similarly carried out.

***In vitro* phosphorylation of Rv2623**

Equimolar concentrations of PknG (one of the Mycobacterial STPK’s (a kind gift of Dr. John Blanchard, Albert Einstein College of Medicine, Bronx, New York) and recombinant Rv2623 (expressed in and purified from either *E.coli* or *M. smegmatis* as described above) were incubated in the presence of 100 μM ATP (Sigma-Aldrich; St. Louis, MO) in kinase reaction buffer (25 mM HEPES pH 7.4, 20 mM MgCl_2_, 20 μM MnCl_2_, 1 mM DTT) at room temperature for different time points. The reaction was stopped by addition of 6X SDS dye and heat inactivating at 65°C for 10 minutes. Samples were electrophoretically resolved in a 10% SDS PAGE, transferred onto a PVDF membrane, and probed with a monoclonal antibody against phosphothreonine (clone #42H4; Cell Signaling; Danvers, MA) and then allowed to react with an appropriate HRP-conjugated secondary antibody. Signals were detected by the Amersham ECL Plus Chemiluminescence kit. Rv2623 protein was identified by molecular mass.

**Thermal denaturation analysis**

Determination of the melting temperature of the Rv2623_WT_ and the Rv2623_T237A_ mutant protein was conducted as previously described [1]. Purified recombinant proteins at 10 μM concentrations (100 mM NaCl; 150 mM HEPES, pH 8.0) were used to determine thermal denaturation. The values were assessed after a 2-hour preincuation at 4^0^C in 5x Sypro Orange protein gel stain (Invitrogen; Carlsbad, CA) using an IQ5 Real Time PCR Detection System (Bio-Rad; Hercules, CA). Increase in fluorescence intensity (λ =575 nm) with an excitation λ of 485 nm for protein samples during a temperature ramp from 20^0^C to 95^0^C was measured. The change in fluorescence reflects quantitative binding of the Sypro dye to the hydrophobic surfaces of the protein under study that are exposed as a result of temperature-dependent protein denaturation. The structural stability of the samples was assessed by the Tm (transition unfolding temperature) that is the first derivative maximum of the fluorescence intensity-temperature curve.

**Mass spectrometry analysis of Rv2623 phosphorylation**

Progenesis was used for the processing of data generated by LCMS as described [5]. The experimental run (+PiB) was aligned chromatographically to that of the control run to minimize the variability of retention time (RT) between the two sets of data. The *m/z* dimension did not required adjustment because of the spectrometer’s high mass accuracy, which is typically less than 3ppm. Standard deviation of Signal:Noise ratio of 3 was set as default for peak detection. Differences in sample loads between injections and noise influence in the data for each run were accounted for using a normalization global scaling factor. To ensure that the best-quality MS-MS spectra data were used for the assignment of peptides the identification of proteins, only the top ten MS/MS per picked peak were included (MS/MS fragment spectra with rank greater than ten were excluded) [5]. An in-house Mascot algorithm (version 2.2.0) [6] was used for database search based on exported MS-MS data (*.mgf*; Mascot generic file) to analyze uninterpreted MS-MS spectra (in Mascot-compatible files generated by the Mascot Distiller program). The parameters used for the Mascot search were those defined previously (partial methionine oxidation, carboxamido-methylated cysteine, peptide and MS-MS fragment tolerance, peptide charges, and number of missed cleavages) [6]. Swiss-Prot protein database (normal and decoy) searches were conducted, using GluC and trypsin as the enzymes. A molecular weight search (MOWSE) score was used to generate the Mascot significance score match, which was based on multiple matches to more than one peptide from the same protein. An *.xml* file of the Mascot search results was generated and using the Progenesis LCMS software, search hits were paired with the LCMS quantitation on the peptide mass.

**References for S1Text**

1. Drumm JE, Mi K, Bilder P, Sun M, Lim J, et al. (2009) Mycobacterium tuberculosis universal stress protein Rv2623 regulates bacillary growth by ATP-Binding: requirement for establishing chronic persistent infection. PLoS Pathog 5: e1000460.

2. Bhatt A, Jacobs WR, Jr. (2009) Gene essentiality testing in mycobacterium smegmatis using specialized transduction. Methods Mol Biol 465: 325-336.

3. Larsen MH, Biermann K, Tandberg S, Hsu T, Jacobs WR, Jr. (2007) Genetic Manipulation of Mycobacterium tuberculosis. Curr Protoc Microbiol Chapter 10: Unit 10A 12.

4. Stols L, Gu M, Dieckman L, Raffen R, Collart FR, et al. (2002) A new vector for high-throughput, ligation-independent cloning encoding a tobacco etch virus protease cleavage site. Protein Expr Purif 25: 8-15.

5. Krishnan N, Lam TT, Fritz A, Rempinski D, O'Loughlin K, et al. (2012) The prolyl isomerase Pin1 targets stem-loop binding protein (SLBP) to dissociate the SLBP-histone mRNA complex linking histone mRNA decay with SLBP ubiquitination. Mol Cell Biol 32: 4306-4322.

6. Hirosawa M, Hoshida M, Ishikawa M, Toya T (1993) MASCOT: multiple alignment system for protein sequences based on three-way dynamic programming. Comput Appl Biosci 9: 161-167.
