# Supplementary material for: Mycobacterium tuberculosis universal stress protein Rv2623 interacts with the putative ATP binding cassette (ABC) transporter Rv1747 to regulate mycobacterial growth
Source: PLoS Pathog. 2017 Jul 28;13(7):e1006515. doi: 10.1371/journal.ppat.1006515 (PMC5549992; doi:10.1371/journal.ppat.1006515)
Supplement: S1 Fig — (DOCX) [file ppat.1006515.s002.docx]

**Supporting Information:**

**S1 Fig**


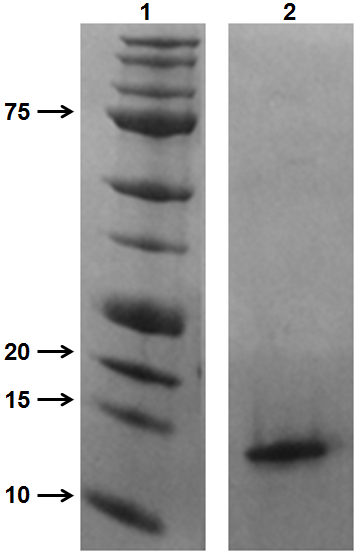


**S1 Fig. Purification of recombinant *M. tuberculosis* Rv1747 FHA I domain.** The cMyc-tagged FHA I domain (1-120 amino acids) of Rv1747 was expressed in the LIC (Ligation Independent Cloning) vector pMCSG7, expressed in *E. coli* , purified, subjected to 10% SDS PAGE electrophoresis and Coomassie Blue-stained (Left) as described in Materials and Methods. Lane 1: Marker; Lane 2: purified Myc-tagged Rv177 FHA I domain (5 μg). A parallel gel was probed with anti-cMyc to validate the assignment of the band as the Rv1747 FHA I domain. Arrows: molecular mass of the corresponding marker band.
