# Supplementary material for: Mycobacterium tuberculosis universal stress protein Rv2623 interacts with the putative ATP binding cassette (ABC) transporter Rv1747 to regulate mycobacterial growth
Source: PLoS Pathog. 2017 Jul 28;13(7):e1006515. doi: 10.1371/journal.ppat.1006515 (PMC5549992; doi:10.1371/journal.ppat.1006515)
Supplement: S2 Fig — (DOCX) [file ppat.1006515.s003.docx]

**Supporting Information:**

**S2 Fig**


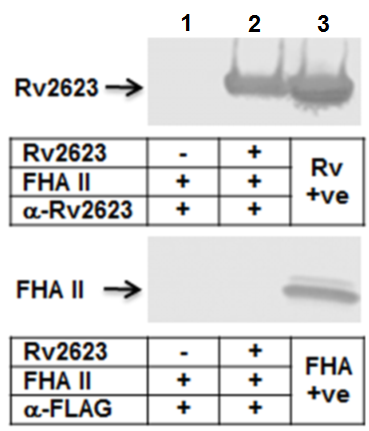


**S2 Fig. *M. tuberculosis* Rv2623 does not interact with Rv1747 FHA II domain: The Co-IP study.** Purified histidine (His_6_)-tagged Rv2623 (Rv2623) and FLAG-tagged FHA II (FHA II: 200-300 amino acids of Rv1747) were expressed in *M. smegmatis* mc^2^155. Purified FLAG-tagged FHA II was passed over columns with or without Rv2623 immobilized onto the Nickle (Ni)-NTA resin. Western analyses of the appropriate elution fractions using anti-Rv2623 and anti-FLAG antibodies (Abs) revealed that Rv2623 and Rv1747 FHA II did not co-elute - upper and lower panels of lane 2 represent the results of probing eluents from column containing both (Ni)-NTA resin-immobilized (His_6_)-tagged Rv2623 and FLAG-tagged Rv1747 FHA II with anti-Rv2623 and anti-FLAG, respectively, thus demonstrating these two mycobacterial components do not interact. Lane 1: upper panel and lower panels represent results of probing eluents from column containing FHA II without immobilized His_6_-tagged Rv2623 with the appropriate Abs. Lane 3 of upper and lower panels represent recombinant Rv2623 (+ve Rv) and FLAG-FHA II (+FHA); respectively, loaded as positive controls. α-Rv2623 and α-FLAG: anti-Rv2623 and anti-FLAG antibodies; respectively.
