# Supplementary material for: Mycobacterium tuberculosis universal stress protein Rv2623 interacts with the putative ATP binding cassette (ABC) transporter Rv1747 to regulate mycobacterial growth
Source: PLoS Pathog. 2017 Jul 28;13(7):e1006515. doi: 10.1371/journal.ppat.1006515 (PMC5549992; doi:10.1371/journal.ppat.1006515)
Supplement: S3 Fig — (DOCX) [file ppat.1006515.s004.docx]

**Supporting Information:**

**S3 Fig**

**
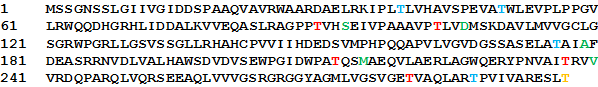
**

**S3 Fig. The Threonine residues of *M. tuberculosis* Rv2623.** The 10 threonine residues of the universal stress protein are colored. Blue depicts residues that are predicted to be not solvent accessible. Red represents solvent accessible residues. Orange designates the threonine that is at the very end of the C-terminus and was not analyzed for solvent accessibility. Green pT+3 residues are those that confer selective interaction with known FHA domains (see Fig 2 of main text and [1-3]).

**REFERENCES**

1. Durocher D, Jackson SP (2002) The FHA domain. FEBS Lett 513: 58-66.

2. Durocher D, Taylor IA, Sarbassova D, Haire LF, Westcott SL, et al. (2000) The molecular basis of FHA domain:phosphopeptide binding specificity and implications for phospho-dependent signaling mechanisms. Mol Cell 6: 1169-1182.

3. Liao H, Yuan C, Su MI, Yongkiettrakul S, Qin D, et al. (2000) Structure of the FHA1 domain of yeast Rad53 and identification of binding sites for both FHA1 and its target protein Rad9. J Mol Biol 304: 941-951.
