# Supplementary material for: Mycobacterium tuberculosis universal stress protein Rv2623 interacts with the putative ATP binding cassette (ABC) transporter Rv1747 to regulate mycobacterial growth
Source: PLoS Pathog. 2017 Jul 28;13(7):e1006515. doi: 10.1371/journal.ppat.1006515 (PMC5549992; doi:10.1371/journal.ppat.1006515)
Supplement: S4 Fig — (DOCX) [file ppat.1006515.s005.docx]

**Supporting Information:**

**S4 Fig**


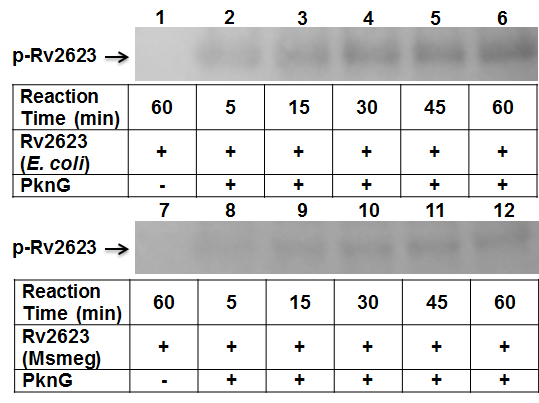


**S4 Fig. *In vitro* phosphorylation of *M. tuberculosis* Rv2623.** Rv2623 was expressed in *E. coli* (upper panel) or *M. smegmatis* (bottom panel), and subjected to in vitro phosphorylation by *M. tuberculosis* PknG as described in Materials and Methods. Reactions made up of recombinant Rv2623 without PknG serves as controls (Lanes 1 and 7). At various times after initiation of the kinasing reaction (from 5 minutes to 60 minutes), the reaction was stopped as described in Materials and Methods. Data depicted represent Western blot analysis of the eletrophoretically resolved (10% SDS-PAGE) reactions upon transfer to PVDF membrane using a monoclonal antibody (clone #42H4) and then allowed to react with an appropriate HRP-conjugated secondary antibody. Signals were detected by the Amersham ECL Plus Chemiluminescence kit. In parallel, reactions were similarly resolved in separate gels and Rv2623 proteins were identified by molecular mass and/or Western blot analysis using antibody against His tag.
