# Supplementary material for: Mycobacterium tuberculosis universal stress protein Rv2623 interacts with the putative ATP binding cassette (ABC) transporter Rv1747 to regulate mycobacterial growth
Source: PLoS Pathog. 2017 Jul 28;13(7):e1006515. doi: 10.1371/journal.ppat.1006515 (PMC5549992; doi:10.1371/journal.ppat.1006515)
Supplement: S5 Fig — (DOCX) [file ppat.1006515.s006.docx]

**Supporting Information:**

**S5 Fig**


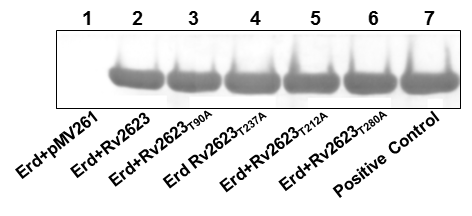


**S5 Fig. The levels of overexpression of Rv2623 protein and its various threonine mutants in *M. tuberculosis* Erdman are comparable.** Wild-type Rv2623 protein and its various T→A mutants were overexpressed in *M. tuberculosis* Erdman via pMV261.

Erd: *M. tuberculosis* Erdman; Erd+pMV261: Erd transformed with vector pMV261 containing no Rv2623 constructs (negative control). Erd+Rv2623, Erd+Rv2623_T90A_, Erd+Rv2623_T237A_, Erd+Rv2623_T212A_ and Erd+Rv2623_T280A_: Erd transformed with pMV261 harboring WT Rv2623, the Rv2623_T90A_, and Rv2623_T237A_, Rv2623_T212A_ and Rv2623_T280A_; respectively. Positive is recombinant His_6_-tagged Rv2623. *M. tuberculosis* Erdman was transformed with pMV261 plasmids expressing His_6_-tagged Rv2623 and the various threonine mutants and the levels of expression of the USP and its variants analyzed by Western blot of proteins obtained from stationary phase bacteria using an Anti-His Ab.
