# Supplementary material for: Mycobacterium tuberculosis universal stress protein Rv2623 interacts with the putative ATP binding cassette (ABC) transporter Rv1747 to regulate mycobacterial growth
Source: PLoS Pathog. 2017 Jul 28;13(7):e1006515. doi: 10.1371/journal.ppat.1006515 (PMC5549992; doi:10.1371/journal.ppat.1006515)
Supplement: S6 Fig — (DOCX) [file ppat.1006515.s007.docx]

**Supporting Information:**

**S6 Fig**

**
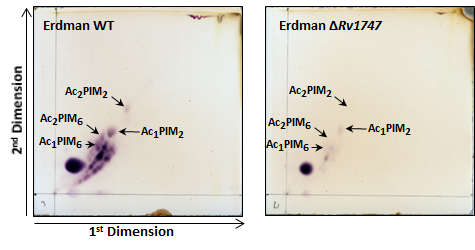
**

**S6 Fig. Analysis of *M. tuberculosis* Erdman WT and Erdman ∆*Rv1747* lipid extracts by 2-dimensional thin layer chromatography.** Lipid extracts were analyzed using protocols similar to that employed for the study of *M. tuberculosis* ∆*Rv2623* (Fig 6E; and see Materials and Methods) except that the first and second dimensions were run in reversed order. The results revealed that the levels of PIMs produced by *M. tuberculosis* Erdman ∆*Rv1747* are less than that by wild-type bacilli (WT).
