# Supplementary material for: Mycobacterium tuberculosis universal stress protein Rv2623 interacts with the putative ATP binding cassette (ABC) transporter Rv1747 to regulate mycobacterial growth
Source: PLoS Pathog. 2017 Jul 28;13(7):e1006515. doi: 10.1371/journal.ppat.1006515 (PMC5549992; doi:10.1371/journal.ppat.1006515)
Supplement: S7 Fig — (DOCX) [file ppat.1006515.s008.docx]

**Supporting Information:**

**S7 Fig**

**S7 Fig. Alignment of *M. tuberculosis* FHA domain-containing proteins.** *M. tuberculosis* has seven protein annotated to contain FHA domains. Of these, only Rv1747 harbors two FHA domains. Black asterisk: complete conserved residue; red asterisk: residues that exhibit 100% identify among all seven FHA-containing proteins except for Rv3360. Alignment was carried out using MUSCLE.
