# Supplementary material for: Mycobacterium tuberculosis universal stress protein Rv2623 interacts with the putative ATP binding cassette (ABC) transporter Rv1747 to regulate mycobacterial growth
Source: PLoS Pathog. 2017 Jul 28;13(7):e1006515. doi: 10.1371/journal.ppat.1006515 (PMC5549992; doi:10.1371/journal.ppat.1006515)
Supplement: S8 Fig — (DOCX) [file ppat.1006515.s009.docx]

**Supporting Information:**

**S8 Fig**

**
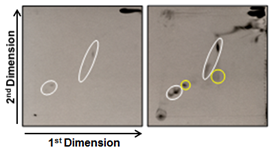
**

**S8 Fig.** **Intracellular *M. tuberculosis* upregulates PIM production.** **Right:** Human macrophage monolayers (1.5 x10^7^) were infected with live ^3^H-^14^C-double labeled *M. tuberculosis* (MOI; 10:1) for 2 h, washed and incubated for an additional 48 h. Infected monolayers were then fixed, lysed, and extracted with organic solvents. **Left:** In parallel, culture-grown bacilli (1.5x10^8^) were similarly processed. Organic solvent extractions were analyzed by 2D-TLC. Autoradiograms showed production of PIMs (white circles) and spots indicating the production of new PIM species or lipoglycan metabolites (yellow circles).
