# Supplementary material for: Mycobacterium tuberculosis universal stress protein Rv2623 interacts with the putative ATP binding cassette (ABC) transporter Rv1747 to regulate mycobacterial growth
Source: PLoS Pathog. 2017 Jul 28;13(7):e1006515. doi: 10.1371/journal.ppat.1006515 (PMC5549992; doi:10.1371/journal.ppat.1006515)
Supplement: S9 Fig — (DOCX) [file ppat.1006515.s010.docx]

**Supporting Information:**

**S9 Fig**


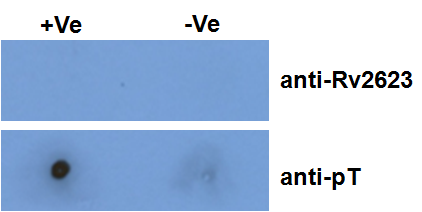


**S9 Fig. Specificity of the antibodies used in the dot-blot assay.** The dot blot analysis was carried out as described in the Materials and Methods section. The membrane was probed with a monoclonal antibody against Rv2623 (anti-Rv2623: Advanced Immunochemicals, Inc., Long Beach, CA; 5-Rv2623-A10) **(Upper Panel)** or with anti-phosphothreonine antibodies (anti-pT: clone #42H4 mouse monoclonal or rabbit polyclonal antibodies (Cell Signaling Technology; Danvers, MA) **(Lower Panel)**. +Ve: 1 μg of positive control for phosphothreonines (Sigma)**;** -Ve: 1 μg of negative control (soybean trypsin inhibitor; Sigma).
